# ChIP-PED enhances the analysis of ChIP-seq and ChIP-chip data

**DOI:** 10.1093/bioinformatics/btt108

**Published:** 2013-03-01

**Authors:** George Wu, Jason T. Yustein, Matthew N. McCall, Michael Zilliox, Rafael A. Irizarry, Karen Zeller, Chi V. Dang, Hongkai Ji

**Affiliations:** ^1^Department of Biostatistics, Johns Hopkins University Bloomberg School of Public Health, Baltimore, MD 21205, ^2^Department of Pediatrics, Texas Children’s Cancer Center, Baylor College of Medicine, Houston, TX 77030, ^3^Department of Biostatistics and Computational Biology, University of Rochester, Rochester, NY 14611, ^4^Department of Microbiology and Immunology, Emory University School of Medicine, Atlanta, GA 30322, ^5^Department of Medicine, Johns Hopkins University School of Medicine, Baltimore, MD 21205 and ^6^Abramson Cancer Center, University of Pennsylvania, Philadelphia, PA 19104, USA

## Abstract

**Motivation:** Although chromatin immunoprecipitation coupled with high-throughput sequencing (ChIP-seq) or tiling array hybridization (ChIP-chip) is increasingly used to map genome-wide–binding sites of transcription factors (TFs), it still remains difficult to generate a quality ChIPx (i.e. ChIP-seq or ChIP-chip) dataset because of the tremendous amount of effort required to develop effective antibodies and efficient protocols. Moreover, most laboratories are unable to easily obtain ChIPx data for one or more TF(s) in more than a handful of biological contexts. Thus, standard ChIPx analyses primarily focus on analyzing data from one experiment, and the discoveries are restricted to a specific biological context.

**Results:** We propose to enrich this existing data analysis paradigm by developing a novel approach, ChIP-PED, which superimposes ChIPx data on large amounts of publicly available human and mouse gene expression data containing a diverse collection of cell types, tissues and disease conditions to discover new biological contexts with potential TF regulatory activities. We demonstrate ChIP-PED using a number of examples, including a novel discovery that *MYC*, a human TF, plays an important functional role in pediatric Ewing sarcoma cell lines. These examples show that ChIP-PED increases the value of ChIPx data by allowing one to expand the scope of possible discoveries made from a ChIPx experiment.

**Availability:** http://www.biostat.jhsph.edu/∼gewu/ChIPPED/

**Contact:** hji@jhsph.edu

**Supplementary information:** Supplementary data are available at *Bioinformatics* online.

## 1 INTRODUCTION

ChIPx experiments, including ChIP-seq (Johnson *et al.*, 2007) and ChIP-chip (Ren *et al.*, 2000), have become a powerful tool used by individual investigators, as well as consortium projects, such as the ENCODE (Dunham *et al.*, 2012) to study transcription factor-binding sites. Each individual ChIPx experiment is non-trivial to perform—extensive time and effort must be spent to acquire effective antibodies and design efficient protocols to generate high-quality ChIPx data—thus, it is important to develop methodology to help investigators to maximize the value of each individual ChIPx experiment.

One of the primary limitations of ChIPx is it may be difficult for individual laboratories to study TF regulation in a wide variety of biological contexts, which we define as the cell or tissue types and associated treatments or disease conditions (see definition details in Supplementary Method 1.1). This is largely because of the prohibitively high labor and time costs to perform each experiment. To resolve this limitation, we investigate whether publicly available gene expression data (PED) in the Gene Expression Omnibus (GEO; Barrett *et al.*, 2009) can be used as a tool to increase the value of ChIPx experiments. Currently, >600 000 gene expression samples from a broad spectrum of biological contexts and species are deposited in the GEO and ArrayExpress (Parkinson *et al.,* 2011). These data are freely available and contain rich information complementary to ChIPx, which may be extremely useful to help study TF regulation.

**Fig. 1.**
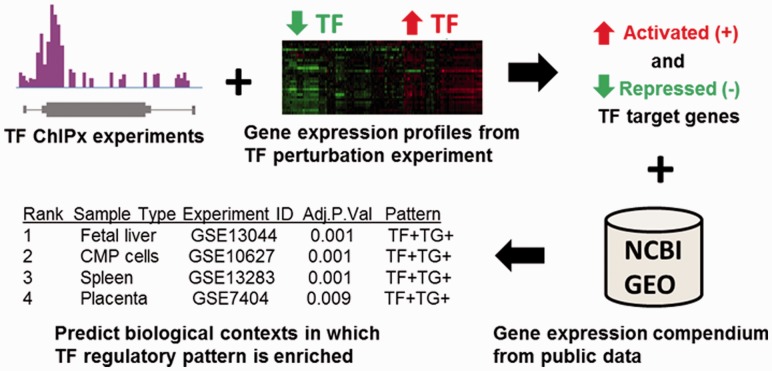
ChIP-PED overview. Gene expression profiles from TF perturbation experiments are intersected with ChIPx experiments to obtain a set of activated and repressed target genes. ChIP-PED then takes as input the TF and target genes and scans through a compendium of publicly available gene expression profiles to search for biological contexts in which the TF and target genes are enriched in activity. The final output is a ranked table of biological contexts enriched with a regulatory pattern of interest

In this article, we demonstrate that this is indeed the case by proposing and evaluating a new approach, ChIP-PED. Given a TF regulatory pathway, i.e. a TF and the corresponding set of target genes defined using ChIPx and gene expression data in one or more biological contexts, ChIP-PED scans through a large collection of >20 000 human and mouse gene expression samples generated by hundreds of different laboratories by quickly surveying the TF and target gene activities across >2000 biological contexts to identify potentially new connections between the TF regulatory pathway and various cell types, tissues or diseases (Fig. 1). We will illustrate that the predictions from ChIP-PED are useful and can greatly expand the scope of discoveries one can make from ChIPx experiments. We also provide an R package for users to perform ChIP-PED analyses on their own ChIPx and TF perturbation data.

ChIP-PED represents a novel conceptual approach to building computational tools for ChIPx data analysis. Most existing tools for analyzing ChIPx data, including those for detecting protein–DNA–binding sites (Laajala *et al.,* 2009; Wilbanks and Facciotti, 2011), discovering DNA-binding motifs (Bailey *et al.,* 2011; Liu *et al.,* 2002), correlating ChIPx with gene expression data (Cheng *et al.,* 2011; Ouyang *et al.*, 2009) and so forth, focus on addressing analysis issues concerning a single or a few related ChIPx datasets. Their discoveries are also typically restricted to the biological context in which the ChIPx experiments are performed, and none of them systematically integrates information from PED. PED has been shown to be invaluable in other applications (Huang *et al.*, 2010; Zilliox and Irizarry*,* 2007), but the possibility of using PED as a tool to boost the analysis of ChIPx data still remains largely unexplored. A number of methods do integrate large amounts of ChIPx and gene expression data to construct gene regulatory networks, but most are primarily used to study lower organisms (e.g. yeast; Faith *et al.*, 2007; Zhu *et al.*, 2008). The present study is different from those described works, as ChIP-PED focuses specifically on integrating ChIPx with large amounts of heterogeneous data in human and mouse to improve ChIPx analyses. Instead of attempting to construct a comprehensive gene regulatory network, the primary goal of ChIP-PED is to produce simple testable hypotheses, such as ‘TF A is functionally active in biological contexts X, Y and Z through target gene set S’.

## 2 MATERIALS AND METHODS

### 2.1 Data collection

ChIP-PED relies on two large compendiums of gene expression profiles, consisting of 13 182 human gene expression samples generated from Affymetrix Human U133A (GPL96) and 9643 mouse samples generated from Affymetrix Mouse 430 2.0 (GPL1261) arrays (McCall *et al.*, 2011). The gene expression profiles were downloaded from GEO (July 2010), pre-processed and normalized consistently using fRMA (McCall *et al.*, 2010). fRMA is designed to normalize large amount of heterogeneous microarray samples to reduce the effect of batch on gene expression estimates. For each probeset, we standardized the fRMA values across all microarray samples from the same array platform to have zero mean and unit standard deviation. The biological context of each sample was recorded and manually verified based on the sample descriptions in GEO (see Supplementary Method 1.1 and Supplementary Fig. S1).

### 2.2 ChIP-PED

Given a TF and its activated and repressed target genes defined using ChIPx and gene expression data in one or more biological contexts, ChIP-PED searches for other contexts in which the TF is likely to be functionally active. Target genes (TG) are genes that are both TF-bound in the ChIPx experiments and differentially expressed in corresponding gene expression data in which the expression of the TF is perturbed. The latter are from TF perturbation experiments comparing wild-type with TF-knockout, control with TF-knockdown or control with TF-overexpression and so forth. Users will need to provide and analyze their own ChIPx and TF perturbation experiments to define the input target genes. Supplementary Method 1.2 discusses methods for generating target gene lists. To define target genes in a particular biological context, ideally one would like to have ChIPx and TF perturbation data from the same biological context. However, such data may not always be available, and it is not uncommon to have ChIPx and TF perturbation data collected from two different contexts. In that case, one can still intersect the data from different experiments to obtain a putative target gene set assumed to contain the shared targets.

ChIP-PED first measures the TF expression and TG activity in each microarray sample in our PED compendiums. TF expression, *E_TF_*, is defined as a simple average of the normalized probeset intensities, *p*: (1)
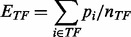
 where *TF* is the set of probesets that measure the expression of the TF, and *n_TF_* is the number of probesets for the TF. TG activity, *A_TG_*, is defined as: (2)
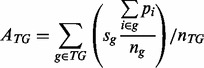



Here, *TG* is the set of target genes of the TF, *n_TG_* is the number of target genes, *n_g_* is the number of probesets for a specific target gene, *g*, and *s_g_* is 1 or −1 depending on whether gene *g* is activated (positively regulated) or repressed (negatively regulated), respectively. *s_g_* is included to account for TFs that are capable of both activating and repressing different target genes. *A_TG_* is designed to describe the regulatory activity of a TF through its target genes, rather than measure the raw expression of the target genes. For example, if a TF acts mainly as a repressor in a biological context in which it is functionally active, we would observe low expression of its target genes, but high *A_TG_* because of the multiplier *s_g_* = −1 (Supplementary Method 1.3 and Supplementary Fig. S2A and B). Examples of the distributions of *E_TF_* and *A_TG_* for real TF ChIPx data are shown in Supplementary Figure S3.

**Fig. 2.**
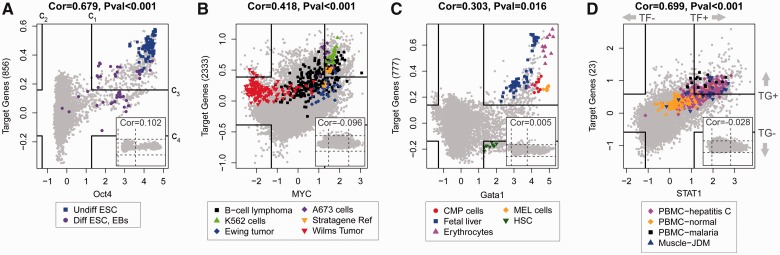
ChIP-PED plots show strong correlation between TF expression, *E_TF_* (*x*-axis) and TG activity, *A_TG_* (*y*-axis), for mouse *Oct4* (**A**) and *Gata1* (**C**) in 9643 Affymetrix Mouse 430 2.0 array samples and human *MYC* (**B**) and *STAT1* (**D**) in 13 182 Affymetrix Human HGU133a samples. Number of TGs in each plot is shown in the parentheses. Solid lines correspond to TF+ (*c_1_*) and TF− (*c_2_*) *E_TF_* cut-offs and TG+ (*c_3_*) and TG− (*c_4_*) *A_TG_* cut-offs. Samples from a few biological contexts with enriched TF+TG+ (in A–D), TF−TG+ (in B) and TF+TG− (in C) functional activity are shown in color. Also plotted in color are ‘Diff ESC, EBs’ (purple) to show the separation between differentiated and undifferentiated ESCs in (A), ‘A673’ (purple) and ‘Ewing tumor’ (blue), both of which are Ewing tumor samples in (B), and ‘PBMC-normal’ (orange), which fall outside of the TF+TG+ region in contrast to infected PBMCs in (D). All other samples are plotted in gray. ‘Cor’: Pearson correlation coefficient between *E_TF_* and *A_TG_*. ‘Pval’: *P*-values that are empirically calculated from *E_TF_* and *A_TG_* correlations of randomly drawn pseudo-TG sets of the same size 10 000 times. For comparison, an example plot of a random sample of pseudo-TGs is shown for each TF (bottom-right)

After measuring TF expression and TG activity, users can choose cut-offs *c_1_ ∼ c_4_* to define (i) high-TF expression (‘*E_TF_ > c_1_*’), (ii) low-TF expression (*‘E_TF_ < c_2_’*), (iii) high-TG activity (*‘A_TG_ > c_3_’*) and (iv) low-TG activity (*‘A_TG_ < c_4_’*), denoted by TF+, TF−, TG+ and TG−, respectively. By default, *c_1_ ∼ c_4_* are chosen to be values corresponding to a one-sided *P-*value of 0.1 based on fitted normal distributions for *E_TF_* or *A_TG_* across all samples, with *c_1_* and *c_3_* taking values above the mean, and *c_2_* and *c_4_* taking values below the mean (Fig. 2A). ChIP-PED can then search for biological contexts associated with four regulatory patterns: (i) TF+TG+, (ii) TF+TG−, (iii) TF−TG+ and (iv) TF−TG−. The pattern TF+TG+ is of primary interest, as it focuses on discovering new contexts in which the TF is functionally active through its target genes (TF-active). This is because high-TF expression alone is not sufficient to imply the existence of functional TF protein because of possible post-transcriptional and translational regulation, but high-TG activity in addition to high-TF expression would strongly support the presence of active TF protein. Other regulatory patterns are discussed in more detail in the Supplementary Method 1.4.

Then given a compendium of *N* gene expression profiles, ChIP-PED searches among all biological contexts with at least three samples, for contexts that are associated with the regulatory pattern of interest (e.g. TF+TG+). For each context *c*, it counts (i) *K*, the total number of samples in the compendium that exhibit the pattern, (ii) *n_C_*, the total number of samples in context *c*, and (iii) *k_C_*, the number of samples in context *c* that exhibit the pattern. Fisher’s exact test is then applied to the quadruplet (*n_C_, N, k_C_* and *K*) to test the association between *c* and the regulatory pattern of interest (i.e. whether *k_C_* is significantly larger than random expectation). To account for testing multiple contexts, the *P*-values are adjusted using the Bonferroni correction. The final output of ChIP-PED is a ranked table of statistically significant biological contexts at a default Bonferroni corrected *P*-value cut-off of 0.05 (Supplementary Method 1.5).

After the initial ChIP-PED analysis, ChIP-PED can perform the following analyses to further explore each predicted context: (i) search for related contexts in the compendium based on user-specified keyword(s), (ii) extract the *E_TF_* and *A_TG_* values for the set of contexts found, (iii) calculate, sort and plot the mean and standard deviation of the *E_TF_* and *A_TG_* values for each context and (iv) perform *t*-tests between all pairwise combinations of the contexts for significant differences in mean *E_TF_* or *A_TG_*. See Supplementary Methods 1.6–1.7 for details and Section 3.3 for an example analysis.

### 2.3 ChIP-PED evaluation

We evaluated ChIP-PED by applying it to multiple TFs—*Oct4, Gata1* and *Jarid2* in mice and *MYC, STAT1* and *ESR1* in human—using the datasets listed in Supplementary Table S1. The TF target genes were constructed by intersecting TF-bound genes predicted from ChIPx data with differentially expressed genes [false discovery rate (FDR) ≤ 10%] in TF perturbation data. TF-bound genes were defined as genes with a significant peak (FDR ≤ 10%) overlapping with the −10- to +5-kb region around the transcription start site of the gene. Details are provided in Supplementary Method 1.2, and full target gene lists can be found in Supplementary Tables S2–S7.

Predictions were verified by a thorough search of existing literature to identify whether each prediction was functionally validated or suggested in previous experiments. ‘Functional’ validations required previous experimental data from the predicted biological context demonstrating observable changes in phenotype when the expression of the TF is perturbed or TF binding coupled with transcriptional responses to TF binding of target genes. ‘Suggested’ predictions must be supported by other lines of indirect evidence, such as experimentally observed high-TF protein levels in the predicted context. All supporting references are recorded in Supplementary Tables S2–S7. We also experimentally validated a novel ChIP-PED functional connection between MYC and Ewing sarcoma (Supplementary Method 1.8).

## 3 RESULTS

### 3.1 PED are capable of measuring TF regulatory activities in spite of data heterogeneity

We first investigated whether it was appropriate to compare gene expression across thousands of heterogeneous microarray samples generated by different laboratories. To this end, we asked whether laboratory and batch effects were a significant and detrimental source of variation (Leek *et al.*, 2010). Previous efforts have been made using our gene expression compendiums to demonstrate that similar tissue types do cluster together (Zilliox and Irizarry, 2007), and that it is possible to accurately predict tissue types from a single gene expression profile in spite of the laboratory or batch effects (McCall *et al.*, 2011). We reaffirmed these findings by observing that samples from the same tissues from different laboratories were more similar in expression compared with samples from different tissues from the same laboratory (Supplementary Fig. S4 and Supplementary Method 1.9).

We then examined the correlation between TF expression (*E_TF_*) and TG activity (*A_TG_*) for multiple TFs, including mouse *Oct4* and *Gata1* and human *MYC* and *STAT1*. We reasoned that if there were strong laboratory or batch effects that overwhelmed the biological signal, we would observe weak to zero correlation between *E_TF_* and *A_TG_* across the heterogeneous samples. Instead, we found significant correlation between TF expression and TG activity; the Pearson correlation coefficients between *E_TF_* and *A_TG_* for *Oct4*, *MYC, Gata1* and *STAT1* were 0.679, 0.418, 0.303 and 0.699, respectively (*P* < 0.02; Fig. 2). As this observation holds for multiple mouse and human TFs from different microarray platforms (GPL1261 and GPL96), our results suggest that biological variability in the publicly available Affymetrix microarray data is stronger than the laboratory or batch effects. This is consistent with earlier observations made by Lukk *et al.* (2010).

### 3.2 ChIP-PED predicts known TF-active contexts

After verifying that it is meaningful to compare *E_TF_* and *A_TG_* across heterogeneous samples, we asked whether the samples observed with high-TF expression and high-TG activity (TF+TG+) and the biological contexts enriched with a TF+TG+ regulatory pattern were biologically meaningful. In this regard, we performed and evaluated ChIP-PED analyses of six TFs: mouse *Oct4*, *Gata1* and *Jarid2* and human *MYC*, *STAT1* and *ESR1*.

*Oct4* is a master regulator in mouse embryonic stem cells (ESCs). We obtained 519 activated and 337 repressed *Oct4* target genes by combining ChIP-seq data from mouse ESCs with gene expression data from ESCs in which *Oct4* was knocked down via siRNA (Supplementary Tables S1 and S2). Using these target genes as input, *Oct4* target gene activity was plotted against *Oct4* expression after excluding the PED samples used to construct the target genes (Fig. 2A). We found that undifferentiated ESCs clustered together with high-TF expression and high-TG activity. In contrast, differentiated ESCs or embryoid bodies (EBs) had lower TF expression and TG activity. This is consistent with the self-renewal and pluripotency role of *Oct4* in ESCs and its decrease in expression when ESCs differentiate (Chen *et al.*, 2008; Loh *et al.*, 2006).

Of the 9643 mouse samples in the compendium, 480 were labeled as TF+TG+ using the default cut-offs. Among them, 69.2% (332/480) were known *Oct4*-expressing (+*Oct4*) biological contexts—most commonly, undifferentiated ESCs (Niwa *et al.*, 2000), primordial germ cells (Kehler *et al.*, 2004), induced pluripotent stem cells (Wernig *et al.*, 2007) and embryonic carcinomas (Wang and Schultz, 1996)—covering 96.0% (332/346) of all +*Oct4* samples in the compendium. In all, 18.1% (87/480) of the TF+TG+ samples were differentiating ESCs or EBs, and the remaining 12.7% (61/480) were other contexts, such as embryos, mouse embryonic fibroblasts (MEFs) and so forth. The observation that a large proportion (30.8% = 18.1% + 12.7%) of TF+TG+ samples were biological contexts not known to express *Oct4* (-*Oct4*) shows the noisy nature of PED. This makes it challenging to correctly predict whether an individual sample truly exhibits functional TF activity. However, the primary goal of ChIP-PED is not to correctly identify TF-active samples, but to identify TF-active biological contexts. Thus, ChIP-PED takes advantage of the fact that each biological context has multiple samples in the PED compendium to predict TF-active biological contexts by reporting the contexts with a statistically significant proportion of TF+TG+ samples.

In total, ChIP-PED predicted 28 biological contexts were enriched with TF+TG+ activity at a Bonferroni-corrected *P*-value cut-off of 0.05 (Supplementary Table S2). Among these, 89.3% (25/28) were different *+Oct4* contexts, and 10.7% (3/28) were *-Oct4* contexts related to differentiating ESCs and EBs. The 28 statistically enriched contexts covered 47.9% (230/480) of the TF+TG+ samples. These samples were from multiple laboratories (e.g. normal undifferentiated ESCs: 11 experiments), confirming that the observed enrichment in ESCs was unlikely to be caused by experimental artifacts or laboratory or batch effects. More importantly, ChIP-PED filtered out most -*Oct4* biological contexts: 30.8% (148/480) TF+TG+ samples were from -*Oct4* contexts, whereas only 10.7% (3/28) of the TF+TG+-enriched contexts were from -*Oct4* contexts, and among the samples of the 28 TF+TG+-enriched contexts, only 8.7% (20/230) were -*Oct4* samples. Therefore, by integrating information from multiple samples, predictions made at the context level are more accurate than at the sample level.

Next, we analyzed human *MYC*, a TF known to be involved in multiple tumors (Zeller *et al.*, 2003). We identified 1716 activated and 617 repressed target genes from a compilation of eight TF perturbation datasets along with 12 ChIPx datasets (Supplementary Tables S1 and S3). The target genes were required to be differentially expressed in the same direction in ≥50% of the TF perturbation datasets and *MYC*-bound in ≥50% of the ChIPx datasets. The aim was to identify the core *MYC* regulatory target genes that were cell-type independent across multiple *MYC*-active contexts. As expected, *MYC* regulatory activity was significantly enriched in numerous tumor types (Figs 2B and 4A and Supplementary Table S3): 74.7% of the 521 TF+TG+ samples were tumors, which was significantly higher than the background percentage of 46.0% for all 13 182 samples in the human PED compendium (one-sided *P* < 0.001, binomial test). Among these samples, ChIP-PED predicted 33 TF+TG+-enriched biological contexts. Many of the predictions were found to be correct, such as B-cell lymphomas, which have been shown to have functionally active MYC protein (Zeller *et al.*, 2003).

Successful predictions were also made when analyzing mouse *Gata1* target genes from erythroid contexts, mouse *Jarid2* target genes from ESCs, human *STAT1* target genes from HeLaS3 cells and human *ESR1* target genes from estrogen-treated MCF7 cells (Supplementary Tables S1, S4–S7). ChIP-PED found enriched *Gata1* expression and target gene activity in expected biological contexts related to erythrocyte and megakaryocyte development, such as in fetal liver, common myeloid progenitor cells and murine erythroleukemia cells (Fig. 2C; Iwasaki *et al.*, 2003). ChIP-PED also predicted *STAT1* functional activity in peripheral blood mononuclear cells (PBMC) infected with hepatitis C and malaria, consistent with current knowledge of *STAT1* regulatory functions (Fig. 2D; Kim *et al.*, 2008; Taylor *et al.*, 2007). *Jarid2* activity, a known repressor with an essential role in embryonic development, was enriched in expected cell types, such as undifferentiated ESCs and induced pluripotent stem cells (Supplementary Fig. S2A; Landiera and Fisher, 2011). Finally, ChIP-PED correctly predicted *ESR1* functional activity in breast cancer-related cell types, such as MCF7 cells (Supplementary Fig. S2C; Frasor *et al.*, 2009).

For the six TFs analyzed, ChIP-PED made 178 TF+TG+ predictions listed in Supplementary Tables S2–S7 (*Oct4*: 28, *MYC*: 33, *Gata1*: 37, *STAT1*: 12, *Jarid2*: 41 and *ESR1*: 27). To systematically evaluate ChIP-PED prediction accuracy, we examined all predictions through a survey of existing literature. We found that 90 of 178 (50.6%) biological contexts predicted to be enriched with TF+TG+ activity were functionally validated in previous experiments (see Section 2). For example in the *Oct4* analysis, 20 of the 28 predictions were functionally validated, even though 25 of the 28 predictions were known to express *Oct4* RNA (i.e. +*Oct4* as described earlier). This is because functional experiments demonstrating changes in phenotype after perturbing *Oct4* or showing TF binding with associated transcriptional response of target genes could only be found for 20 predictions; therefore, we only counted those 20 predictions as functionally validated. The 50.6% accuracy rate is a conservative estimate, as the remaining predictions may not necessarily be false positives, but instead may represent unknown/novel functional relationships. Altogether, these results demonstrate that given the target genes of a TF defined from ChIPx and TF perturbation data from one or a few biological contexts, ChIP-PED is capable of discovering TF-active contexts from a broad spectrum of PED samples.

Searching through PED only for TF+ samples or only for TG+ samples, rather than TF+ and TG+ samples, may result in substantially decreased ChIP-PED prediction accuracy and number of functionally validated predictions. For instance, when we modified ChIP-PED to search only for TF+ samples, we found that only 40.0% (62/155) of the predicted TF+ contexts were functionally validated in previous experiments compared with 50.6% (90/178) when using ChIP-PED to search for TF+ and TG+ samples (Supplementary Tables S2–S7). Conversely, searching for only TG+ samples resulted in only 34.0% (67/197) functionally validated TG+ predictions (Supplementary Tables S2–S7). Thus, it is useful to check both TF expression and target gene activity of each context to identify TF+ and TG+ samples when predicting TF-active biological contexts.

TF target genes can vary from one cell type to another. If two known TF-active contexts do not share any target genes, then ChIP-PED will not be able to predict either context using target genes constructed from the other context. To test whether ChIP-PED can still be effective when only a minority of the target genes are shared, we used ChIP-PED to analyze *Stat3* target genes constructed from mouse CD4+ T cells and Th17 cells, which are both contexts in which *Stat3* plays an important regulatory role (Durant *et al.*, 2010; Kwon *et al.*, 2009). We found that ChIP-PED was able to successfully recover both CD4+ T cells and Th17 cells when analyzing target genes defined from the other context, even though <30% of the target genes were in common (Supplementary Method 1.10, Supplementary Table S8 and Supplementary Fig. S5).

### 3.3 ChIP-PED can expand the scope of possible functional discoveries

ChIP-PED would not be useful if the predicted biological contexts were always closely related to the context in which the experimental data were generated. Our results indicate otherwise: among the 90 of 178 (50.6%) predictions supported by previous functional experiments, 40 (44.4%) are in contexts unrelated to the context(s) in which the experimental data used to construct the TF target genes were obtained (Supplementary Tables S2–S7).

**Fig. 3.**
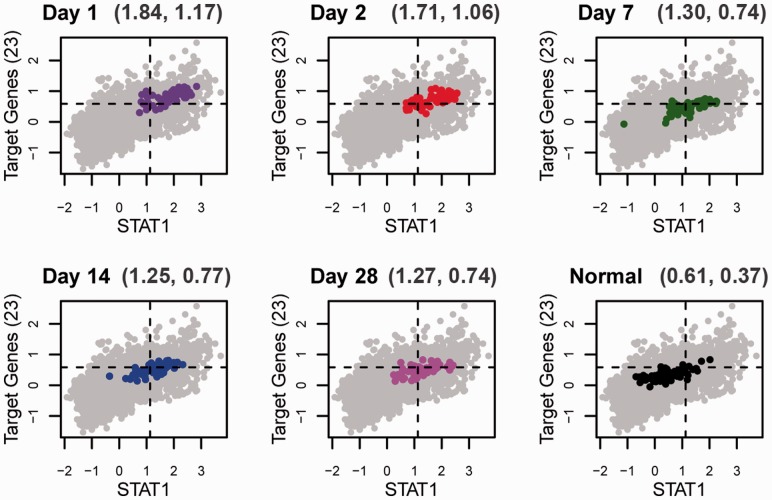
Series of ChIP-PED plots depicting the gradual decrease in *STAT1* expression and target gene (TG) activity when blood samples are successively drawn from hepatitis C-infected patients as they recover after treatment with interferon and ribavirin from Day 1, 2, 7, 14 to 28 of recovery (GSE7123). Gray points are all samples in the GPL96 compendium, and colored points are the samples from the infected PBMCs in GSE7123. The *x*-axis is *STAT1* expression (*E_TF_*) and the *y*-axis is TG activity (*A_TG_*). The mean *E_TF_* and *A_TG_* of each group of PBMCs are indicted at the top of each plot. Normal PBMCs (bottom right in black) in GSE7123 fall almost entirely out of the TF+TG+ cut-offs (the dashed lines), which suggests that only when infected with hepatitis C is *STAT1* functionally active in PBMCs

Furthermore, ChIP-PED can provide additional biological insights that otherwise could not be made using standard ChIPx analyses. For example, after the initial *STAT1* ChIP-PED analysis described in Section 3.2, we found many hepatitis C-infected PBMCs predictions from experiment GSE7123 (Supplementary Table S5 and Supplementary Method 1.11). To examine *STAT1* functional activity in hepatitis C-infected PBMCs in more detail, we searched for all contexts in GSE7123 and also found healthy PBMCs along with the predicted hepatitis C-infected PBMCs. We then used ChIP-PED to compare TF expression and TG activity in each context and found that *E_TF_* and *A_TG_* values were significantly different between healthy and hepatitis C-infected PBMCs, with a gradual decrease in *E_TF_* and *A_TG_* values as patients recovered from infection (Supplementary Table S5, Fig. 3 and Supplementary Fig. S6). When reviewing both of the original publications, the *STAT1* ChIPx study (Robertson *et al.*, 2007) and the study that generated the gene expression profiles from hepatitis C-infected PBMCs (Taylor *et al.*, 2007), we found that neither study had reported this finding. To verify whether this observation was correct, we searched through existing literature and found an entirely independent experiment that showed in a series of overexpression and siRNA-mediated knock-down experiments of *STAT1* in hepatitis C virus-infected PBMCs that *STAT1* protein was indispensable for the control of hepatitis C virus expression (Lin *et al.*, 2005).

### 3.4 ChIP-PED can discover novel TF-active contexts

**Fig. 4.**
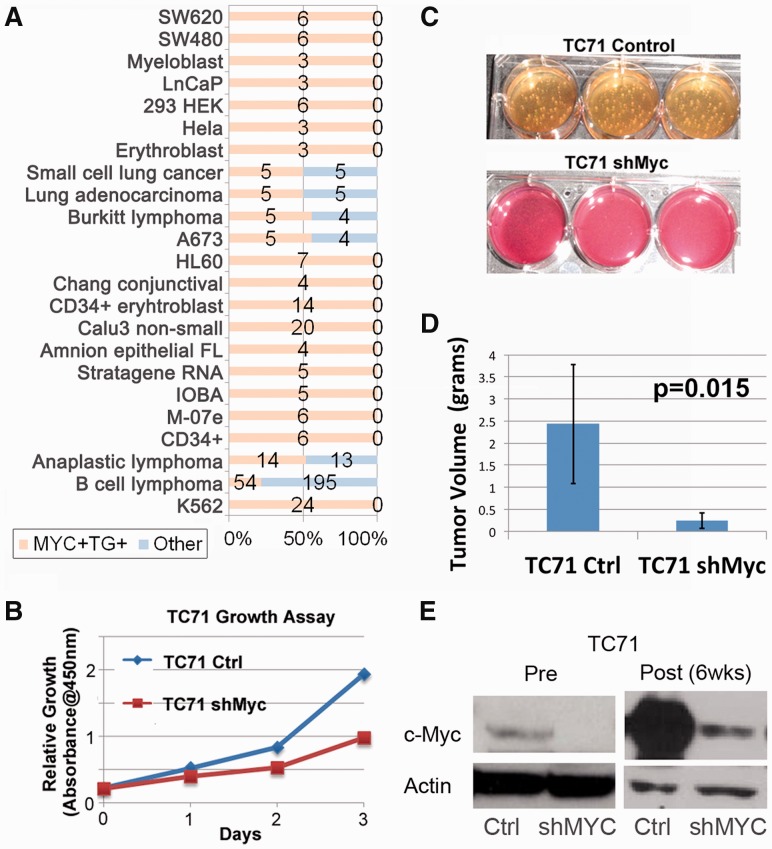
*MYC* analysis and validation. (**A**) *MYC* TF+TG+ biological contexts (similar contexts are grouped) and the number of *MYC*+TG+ samples (orange) and number of non-*MYC*+TG+ samples (blue) are shown. The majority of TF+TG+ samples are tumor types. (**B**) Decrease in proliferation of TC71 cells on knockdown of *MYC*. Control and shMyc TC71 cells were evaluated for changes in proliferation rates by using a cell viability reagent, CCK-8. Two thousand cells were initially plated into individual 96 wells and assessed daily for changes in growth and proliferation. (**C**) Decreased tumorigenicity as assessed by soft-agar assay for shMyc TC71 cells. Control TC71 cells developed significant soft-agar colonies within 2–3 weeks, whereas the shMyc cells formed only a few miniscule colonies over the same time. (**D**) Graphic display of differences in tumor weight comparing control and shMyc tumors. On average, the shMyc tumors weighed only 20% of control tumors. Vertical error bars indicate the standard deviation of the tumor volume. The *P*-value is obtained from a two-sided *t*-test. (**E**) Western blot analysis for MYC protein, c-Myc, in control and shMyc cells at 0 (Pre) and 6 weeks (Post). Actin is provided as a loading control. Blot displays decrease in MYC protein levels on stable expression of shMyc in TC71 cells

Besides verifying that ChIP-PED is able to correctly predict known TF-active biological contexts, we also experimentally investigated whether the predictions that were not functionally validated could possibly represent unknown TF-active biological contexts. As a proof-of-concept, we used our ChIP-PED analysis of human *MYC* to illustrate the discovery of a novel *MYC*-active context. Among the enriched TF+TG+ predictions from the *MYC* ChIP-PED analysis, 18 of 33 (54.5%) biological contexts were not supported by functional experiments that demonstrated *MYC* functional activity (Supplementary Table S3). One of the non-functionally validated contexts was A673 cells (Fig. 4A), which were established from a patient with Ewing sarcoma (Martínez-Ramírez *et al.*, 2003). Although Ewing tumor has been previously shown to exhibit high-*MYC* expression (Dauphinot *et al.*, 2001), the functional role of MYC protein in Ewing tumor currently remains uncharacterized. To verify the novel prediction that MYC protein plays a functional role in Ewing tumor, we assessed the phenotype changes of independent Ewing sarcoma cell lines on *MYC* knockdown. Knocking down of *MYC* using shMYC in TC71 and MHH-ES Ewing sarcoma cell lines resulted in a substantially slower proliferation rate and tumorigenicity when compared with control cells (Fig. 4B and C and Supplementary Figs S7 and S8). Furthermore, xenograft of control and shMYC TC71 Ewing sarcoma cells into immunodeficient mice (NOD/SCID/IL-2γ null) resulted in a significant decrease in volume and weight for the *MYC* knockdown tumors after 6 weeks of growth (Fig. 4D). Subsequent isolation of the tumors confirmed the decrease in MYC protein by western blot analysis (Fig. 4E). These results strongly support the novel prediction that the MYC protein plays a key functional role in Ewing tumor.

When studying the 88 functionally unverified predictions across the six TFs analyzed, we found that 51 of the 88 (58.0%) predictions were supported by other lines of indirect evidence in existing literature, such as experimentally observed high-TF protein level in the predicted context (Supplementary Tables S2–S7). Thus, these predictions are likely to represent previously unknown functional predictions between each TF regulatory pathway and context, which further demonstrates that ChIP-PED can discover known and unknown TF-active biological contexts. In total, 141 of 178 (79.2%) TF+TG+ predictions for the six TFs analyzed were either directly supported by functional evidence (90 of 178) or indirectly supported in existing literature (51 of 178).

### 3.5 Effect of modifications to the ChIP-PED analysis

Many TFs regulate a subset of their target genes through distal enhancers. Recent tools, such as GREAT (McLean *et al.*, 2010), have shown that by properly accounting for distal regulatory sites, one can improve the functional analysis of TF-binding sites. In our ChIP-PED analyses, we assigned peaks to genes if the peak overlapped with the −10- to +5-kb region around each gene transcription start site, which may miss distal TF regulatory activity. This in turn may affect ChIP-PED prediction accuracy. To investigate, we generated ChIP-PED predictions for ESR1 using target genes in estrogen-treated MCF7 cells derived from chromatin interaction analysis by paired-end tag sequencing (ChIA-PET), a method better able to link distal regulatory sites to TF-binding targets (Fullwood *et al.*, 2009), and compared them with predictions made using target genes defined by ChIP-seq using the −10- to +5-kb window. We found that ChIA-PET-based predictions were similar to ChIP-seq–based predictions, and the former had slightly higher functional prediction accuracy of 43.5% compared with 40.7% (Supplementary Method 1.12 and Supplementary Table S7). We also analyzed all six TFs by using multiple annotation window sizes to annotate ChIPx peaks. Different window sizes produced comparable prediction accuracies at the default significance cut-off. However, the −10- to +5-kb window size produced the largest number (i.e. highest power) of functionally validated and/or indirectly supported predictions (Supplementary Method 1.12 and Supplementary Tables S9 and S10). Thus, our results suggest that the −10- to +5-kb window represents a reasonable choice as a default annotation region.

We also compared how well a median, rather than mean, target gene activity measure would perform and found the predictions and prediction accuracy to be almost the same; across all six TFs, 171 predictions were identical between the two measures accounting for 98.8% (171/173) of the median-based predictions and 96.1% (171/178) of the mean-based predictions (Supplementary Method 1.13 and Supplementary Fig. S9). In addition, we checked whether predicted biological contexts with more samples in the compendium were more or less accurate than predicted contexts with fewer samples. Our results were unable to find a clear monotone relationship between sample count for a given biological context and prediction accuracy (Supplementary Method 1.14 and Supplementary Table S11).

## 4 DISCUSSION

We have shown that ChIP-PED can improve the analysis of ChIPx data by integrating publicly available gene expression data. Given a TF and its target genes, ChIP-PED examines the expression of the TF and the activity of its target genes across an assortment of diverse biological contexts to search for contexts with enriched regulatory activity of the TF. This process may lead to the discovery of novel functional connections between TF regulatory pathways and diseases, thus providing a cost effective way to expand knowledge from one ChIPx study to other research areas.

We view ChIP-PED as an exploratory tool for fast and cost-effective hypothesis generation and screening. In this respect, the default cut-offs that define high- or low-TF expression or TG activity should be primarily used for initial exploration or first-pass automatic hypothesis screening, rather than as strict optimal cut-offs that apply to all TFs. Based on our real data analysis experience, we found it difficult to set a single consistent cut-off that was optimal across all TFs, as TFs can vary greatly in terms of regulatory behavior (Fig. 2). We, therefore, provide users with the flexibility to choose their own cut-offs, which can be adjusted to decrease or increase the number of predicted contexts (Supplementary Method 1.15).

ChIP-PED acts primarily as a guide to highlight biological contexts that would be good leads for experimental investigation. As such, we do not expect all ChIP-PED predictions to be correct nor for ChIP-PED to recover all TF-active biological contexts. This, however, does not prevent ChIP-PED from being a useful and unique tool: our analyses have shown that it can predict many known and new TF-active contexts with reasonable accuracy, and there currently exists no other computational method for analyzing ChIPx data that performs a similar task.

Although we have shown that ChIP-PED is able to capture pertinent biological information in PED, better statistical models are still needed to address technical biases and variations because of laboratory and batch effects. A natural extension of ChIP-PED would be to analyze multiple TFs and their TGs together to better connect cooperative TF regulatory pathways to cell types and diseases. Similarly, more work is also needed to understand how homologous TFs or other TFs with similar regulatory functions impact the regulatory activity of the TF of interest in different contexts.

Our study is not necessarily the best or only way to integrate ChIPx and PED; however, to the best of our knowledge, this is the first systematic study of using PED to enhance ChIPx analyses in human and mouse. We hope that ChIP-PED will inspire new computational approaches that continue to maximize the value of ChIP-seq and ChIP-chip experiments.

*Funding*: National Institutes of Health (R01HG005220 and R01HG006282 to R.A.I and H.J.); National Institutes of Health training (T32GM074906 to G.W.); St. Baldrick’s Career Development Award (to J.T.Y.).

*Conflict of Interest*: none declared.

## Supplementary Material

Supplementary Data
