# Supplementary material for: ChIP-PED enhances the analysis of ChIP-seq and ChIP-chip data
Source: Bioinformatics. 2013 Mar 1;29(9):1182–9. doi: 10.1093/bioinformatics/btt108 (PMC3658457; doi:10.1093/bioinformatics/btt108)
Supplement: Supplementary Data [file supp_29_9_1182__index.html]

ChIP-PED enhances the analysis of ChIP-seq and ChIP-chip data — ChIP-PED enhances the analysis of ChIP-seq and ChIP-chip data — ChIP-PED enhances the analysis of ChIP-seq and ChIP-chip data — Supplementary Data 

# ChIP-PED enhances the analysis of ChIP-seq and ChIP-chip data

## Supplementary Data

files

**Files in this Data Supplement:**

- Supplementary Data - docx file
- Supplementary Data - xlsx file
- Supplementary Data - xlsx file
- Supplementary Data - xlsx file
- Supplementary Data - xlsx file
- Supplementary Data - xlsx file
- Supplementary Data - xlsx file
- Supplementary Data - xlsx file
- Supplementary Data - xlsx file
