# Supplementary material for: ChIP-PED enhances the analysis of ChIP-seq and ChIP-chip data
Source: Bioinformatics. 2013 Mar 1;29(9):1182–9. doi: 10.1093/bioinformatics/btt108 (PMC3658457; doi:10.1093/bioinformatics/btt108)
Supplement: Supplementary Data [file supp_btt108_ChIP-PED_Supp_Rev1.7.7.2.docx]

| Category  ChIP-PED (Supplementary Materials)  George Wu^1,#^, Jason T. Yustein^2,#^, Matthew N. McCall^3^, Michael Zilliox^4^, Rafael A. Irizarry^1^, Karen Zeller^5^, Chi V. Dang^6^, and Hongkai Ji^1,*^  ^1^Department of Biostatistics, Johns Hopkins University Bloomberg School of Public Health, Baltimore, MD 21205, USA  ^2^Department of Pediatrics, Texas Children’s Cancer Center, Baylor College of Medicine, Houston, TX 77030, USA  ^3^Department of Biostatistics and Computational Biology, University of Rochester, Rochester, NY 14611, USA  ^4^Department of Microbiology and Immunology, Emory University School of Medicine, Atlanta, GA 30322, USA  ^5^Department of Medicine, Johns Hopkins University School of Medicine, Baltimore, MD 21205, USA  ^6^Abramson Cancer Center, University of Pennsylvania, Philadelphia, PA 19104, USA  ^#^These authors contributed equally to this work.  Received on XXXXX; revised on XXXXX; accepted on XXXXX  Associate Editor: XXXXXXX |
| --- |

# Supplementary methods

## Definitions and compilation of biological contexts

In this article, a ***gene expression profile***, also called a ***gene expression sample***, refers to the measurements of the expression of all genes from a single microarray hybridization. A ***gene expression experiment*** is a group of gene expression samples collected by a lab to study a particular biological question of interest. A ***species*** is a population of organisms with a high level of genetic similarity, e.g. mouse or human. To develop ChIP-PED, gene expression profiles from publicly available gene expression data for two species, human and mouse, were collected. This resulted in two large compendiums of gene expression samples, one for each species.

Within each species, samples are grouped based on biological contexts. Broadly speaking, one can define a ***biological context*** as a common characteristic in terms of the sample origins (e.g., cell type, tissue type, disease condition, treatment, and/or other characteristics, as well as their combinations) shared by a group of samples. In this article, however, we adopt a more technical definition of biological context for the convenience of implementation: we define ***biological context*** as the cell or tissue type and the associated treatment or disease condition of the sample informed by the GEO annotations.

The biological context associated with each gene expression sample in our PED compendiums is acquired from NCBI GEO (Barrett *et al.*, 2009). These context labels were curated by an expert by reading the sample annotation files in GEO. Each gene expression sample in GEO has a text annotation file named as GSM***, where *** is the sample identifier. The gene expression experiment that contains the sample also has an annotation file named as GSE***, where *** is the experiment identifier. These files were downloaded from GEO. Information was first extracted automatically from multiple fields in the GSM files, including the “Title”, “Source name”, “Characteristics”, “Description” and various “Protocol” fields. The expert then read the extracted information to determine the cell or tissue type of each sample, as well as the treatment or disease condition under which the sample was collected. The expert also cross-checked this information with the GSE files associated with the sample. Each sample is then manually annotated based on the cell or tissue type and the associated treatment or disease condition. For example, if the “Characteristics” field says that a gene expression sample is from “MCF7 cells”, and the “Description” field contains “treated with E2”, then the expert will know that the cell type of the sample is MCF7 and the gene expression sample is obtained after applying the E2 treatment. As a result, the biological context of the sample would be annotated as “MCF7 cells: treated with E2”, where a colon, “:”, separates the cell or tissue type from the treatment or disease condition. Since the raw annotation data in GEO are provided by the investigators who originally generated and submitted the gene expression data, the level of specificity of the biological context label of each sample will vary depending on the information given.

Using this approach, a total of 9643 mouse samples from 3144 different mouse biological contexts and 13,182 human samples from 2631 different human biological contexts were annotated. Among these contexts, 1615 mouse and 1867 human biological contexts contained only one or two gene expression samples corresponding to a total of 2291 mouse samples and 2231 human samples (**Figure S1**). 1529 mouse and 764 human biological contexts contained ≥3 expression samples corresponding to a total of 7352 mouse samples and 10,951 human samples (**Figure S1**). To ensure that the significantly enriched biological contexts reported by ChIP-PED are supported by multiple samples, the Fisher’s exact test is only applied to the 2293 (= 1529+764) contexts with ≥3 samples. **Figure S1** provides a general overview of the distribution of sample counts per context by displaying the number of biological contexts with different sample counts.

Although contexts with only one or two sample(s) are not tested for enrichment, they are still included in our compendiums because they contain information to better estimate the background (i.e., the total number of compendium samples, *N*, and the number of samples that exhibit the regulatory pattern of interest, *K*, in the Fisher’s exact test for significant association of the regulatory pattern of interest with a given context). Furthermore, these contexts may also help users to quickly explore new hypotheses. For example, some users may already have a list of hypotheses (e.g., possible contexts involving functional activity of a TF of interest) generated by other types of experimental data and they may only have resources to rigorously test a few of them. Since these hypotheses are obtained based on other experimental data, whether or not ChIP-PED is able to detect significant enrichment of a TF regulatory pattern may not be of importance. Instead, such users may be more interested in how strong TF regulatory activity (e.g. high TF expression and high TG activity) is in different contexts relative to other contexts in the compendium, which may then help them to select the most interesting hypotheses to verify first. In this scenario, PED is used as a secondary filter, and the biological contexts with one or two samples will still be very useful if they are involved in the users’ hypotheses.

In the current implementation of ChIP-PED, the samples are manually annotated based on our technical definition of a biological context. Each gene expression sample is only labeled with one biological context, and no sample is annotated using two or more contexts. Thus, two gene expression samples from the same cell or tissue type but different treatment or disease conditions will be annotated as two different biological contexts. Ideally, we would like to employ a broader biological context definition in ChIP-PED, which allows users to test for enrichment of TF regulatory activity at different levels of specificity (e.g., “stem cell”, “embryonic stem cell”, and “embryonic stem cell treated with drug A”, etc.). In order to do so, this would require a hierarchy of biological context labels for each sample in the compendium. For example, samples from MCF7 cells may be labeled as MCF7, breast cancer cell line, or more generally as diseased. Then users can test for TF regulatory activity in a specific cell type, a tissue type, or more generally in diseased contexts. Building such a hierarchy manually is not feasible due to the prohibitively high manual labor and time costs. Thus, one future research direction to extend ChIP-PED is to investigate methods to automatically annotate the samples using controlled vocabularies. When these methods become available, users will be able to define a group of samples with a common characteristic (e.g., “liver”) or combination of characteristics (e.g., “liver” and “tumor”) as belonging to the same biological context. Then ChIP-PED can be used to test the association between TF regulatory activities and biological contexts defined by the different combinations of controlled vocabularies. This would make the analysis more flexible because biological contexts could then be defined at multiple hierarchical levels and each sample could be annotated with multiple biological contexts. The challenge of this approach, however, is to automatically and accurately annotate the samples by parsing the GEO text annotation files. This is still a non-trivial task and developing such a solution is still under investigation.

In the future, ChIP-PED may also be extended to include PED compendiums compiled from RNA-seq data. We first focused on microarray data because there are 600,000+ gene expression microarray samples compared to 6,000+ RNA-seq samples currently deposited in GEO. The much larger number of microarray samples allows us to test our method across a wider range of biological contexts to better establish an initial proof-of-principle. Furthermore, methods to properly normalize and systematically compile large numbers of RNA-seq data still need to be explored. In comparison, methods to normalize and systematically analyze microarray data are relatively more established.

## Construction of ChIP-PED target genes

ChIP-PED requires users to input the activated and repressed target genes of a TF of interest in order to predict biological contexts enriched with functional activity of the TF. Thus, users will need to collect and analyze both ChIPx and TF perturbation data to define the TF target genes.

In this article, the input TF target genes were constructed by intersecting TF-bound genes from ChIPx experiments with differentially expressed genes from corresponding TF perturbation experiments, using the data listed in **Supplementary Table S1**. For ChIP-seq experiments, peak lists that contained the TF binding sites were directly extracted from the ENCODE website at UCSC (http://genome.ucsc.edu/) or from the original publications. A gene was defined as bound by a TF if a significant peak (FDR ≤ 10%) overlapped with the 10kb upstream to 5kb downstream region around the transcription start site (TSS) of the gene. Gene expression data from TF perturbation experiments were first processed with RMA (Irizarry *et al.,* 2003) then analyzed with limma (Smyth, 2004) to identify differentially expressed genes (FDR ≤ 10%). When multiple ChIPx or expression datasets were analyzed together, target genes had to be differentially expressed in ≥ 50% of the gene perturbation experiments and TF-bound in ≥ 50% of the ChIPx experiments. In addition, we required target genes to be consistently regulated across all TF perturbation experiments, meaning that target genes were not allowed to be both positively and negatively differentially expressed across the TF perturbation experiments. Only for *STAT1* analyses, target genes were compiled by intersecting TF-bound genes from ChIPx data with experimentally validated functional targets from literature (Robertson *et al.*, 2007), rather than using gene perturbation data. All target genes used in this study are listed in **Supplementary Tables S2-S7**.

More generally, for users unfamiliar with the ChIPx and gene expression data analyses, below is a procedure they may use to construct target genes from their ChIPx and gene expression data.

First, users will need to identify and annotate TF binding sites (TFBSs) from ChIPx data. If users want to use public ChIPx data, they could go to databases such as ENCODE (ENCODE Project Consortium *et al.*, 2011), hmChIP (Chen *et al.*, 2011), Cistrome (Liu *et al.*, 2011), ChEA (Lachmann *et al.*, 2010), etc. to search for available datasets and download the binding site lists. If users have their own data, they can analyze the data using any existing ChIPx peak caller. For instance, we usually analyze our ChIPx data using CisGenome (Ji *et al.*, 2008). In the latest version of CisGenome (v2.0), the following functions are provided for processing ChIPx data: (1) ***seqpeak*** for calling peaks from ChIP-seq data with both IP and control samples; (2) ***seqpeak_iponly*** for calling peaks from ChIP-seq data with only IP samples; (3) ***tilemapv2*** for calling peaks from ChIP-chip data using the TileMap algorithm (Ji *et al.*, 2005). One could also use other tools such as MACS (Zhang *et al.*, 2008) for ChIP-seq peak calling and MAT (Johnson *et al.*, 2006) for ChIP-chip peak calling. Information on how to use these tools can be found in their manuals and will not be discussed here. After detecting significant TFBSs in the ChIPx data, ChIP-PED provides an R function, ***annotatePeaks*,** which allows users to associate binding sites to nearby genes by specifying an annotation window size. The input binding site data should be organized such that the first column contains the name of the chromosome (e.g. chr1), the second column contains the start position of the binding site in the chromosome, and the third column contains the end position of the binding site in the chromosome. Additional columns can also be added for other non-required information. Genes associated with the TFBSs are then defined as TF bound genes.

Next, users will need to identify genes that respond transcriptionally to changes in TF expression by detecting differentially expressed genes in gene expression data from TF perturbation experiments. There are a large number of tools available for this purpose. As an example, for analyzing gene expression data from Affymetrix arrays, one can use RMA (Irizarry *et al.,* 2003) to preprocess and normalize the data, and then use limma (Smyth, 2004) to detect differentially expressed genes. Both methods have R packages available for download in Bioconductor (www.bioconductor.org). To analyze Affymetrix exon array data, one could use the GeneBASE software (Kapur *et al.*, 2008) for preprocessing and normalization. The normalized gene expression values can then be analyzed using limma to identify differentially expressed genes. For RNA-seq, one may use tools such as Cufflinks (Trapnell *et al.*, 2012), DESeq (Anders *et al.*, 2010), and/or edgeR (Robinson *et al.*, 2010) for data preprocessing and to identify differentially expressed genes.

Finally, after users have obtained both TF-bound genes from the ChIPx data and differentially expressed genes from the TF perturbation gene expression data, they can use the function, ***ConstructTG***, in the ChIP-PED package to derive the activated and repressed TF target genes required for a ChIP-PED analysis. ***ConstructTG*** is another function in the ChIP-PED package and takes as input a list of annotated peaks derived from ChIPx data (same format as required for ***annotatePeaks***, but with an additional column for peak-to-gene annotations in Entrez Gene ID format labeled as ‘EntrezGeneID’) and a list of annotated differentially expressed genes from TF perturbation data (the analysis summary from the topTable function in limma with an additional column, as the first column, for probe-to-gene annotations). Then, ***ConstructTG*** will intersect the TF-bound and differentially expressed genes to obtain activated and repressed target genes, where activated (repressed) targets are TF-bound and increase (decrease) in expression when the TF expression increases. For more details, a full example of how to construct TF target genes from real ChIPx and TF perturbation data is described on the ChIP-PED webpage.

Construction of the input target genes for ChIP-PED requires one to have both ChIPx data and TF perturbation gene expression data. While the number of ChIPx datasets in public domains is rapidly growing, a quick survey of 58 ChIPx studies randomly chosen from GEO showed that a large fraction (26/58 = 44.8%) of the published ChIPx experiments did not have accompanying TF perturbation data for the same TF in the same biological context. Conversely, we also did a quick survey of 253 TF perturbation experiments in human and mouse in GEO and found that 79.8% (202/253) did not have corresponding ChIPx data for the same TF in the same biological context. Thus, the lack of matching ChIPx and TF perturbation data may limit the applicability of ChIP-PED.

To partially alleviate this issue, one may intersect ChIPx data and TF perturbation data for the same TF but from different biological contexts to construct TF target genes. For instance, in our survey above, while 79.8% (202/253) TF perturbation experiments did not have corresponding ChIPx data for the same TF in the same biological context, this percentage dropped to 68.8% (174/253) if one is willing to use ChIPx data for the same TF but collected from other contexts. Similarly, while 44.8% (26/58) ChIPx data do not have corresponding TF perturbation data, the percentage dropped to 36.2% (21/58) if one is willing to match the ChIPx data with TF perturbation data from another context. When one intersects ChIPx and TF perturbation data from different biological contexts, not all target genes in either biological context will be recovered. However, users would at least be able to obtain a subset of the desired target genes that are assumed to be in common between the two biological contexts.

Since combining ChIPx and TF perturbation data from different contexts most likely will not recover all TF target genes specific to either context, we encourage investigators to perform both ChIPx and accompanying TF perturbation experiments when they study the regulatory behavior of one or more TF(s) in one or more context(s). This would also allow them to use tools such as ChIP-PED to expand the possible functional discoveries by examining the regulatory behavior of their TF(s) across a diverse landscape of public available gene expression data. Furthermore, the generated experimental data could then be deposited in a public database, which may be helpful to other researchers that are also studying regulatory activities of the same TF(s).

## ChIP-PED TF expression and target gene activity measures

The purpose of ChIP-PED is to predict which biological contexts are enriched with the regulatory activity of a TF given the activated and repressed target genes of the TF defined from ChIPx and TF perturbation data in one or more contexts. In order to do so, ChIP-PED relies on two measures - TF expression, *E_TF_*, and target gene activity, *A_TG_*. Here, we will provide some more intuition behind the design of both measures.

TF expression, *E_TF_*, is a straightforward average of the gene expression estimates for the TF using all probesets designed to measure the expression of the TF. Target gene activity, *A_TG_*, on the other hand needs to account for multiple target genes, some of which may be activated (positively regulated) by the TF whereas others may be repressed (negatively regulated). For the positive targets, increased or high expression of TF will increase or activate target gene expression. For the negative targets, increased or high expression of TF will decrease or repress the target gene expression. Both high expression of positive targets and low expression of negative targets are indicators of the regulatory activity of the TF. Accordingly, in formula (2) of the main text, *s_g_* is used to modify the negative expression value for negative target genes (remember the compendium is standardized to have zero mean, and therefore repressed genes usually have negative expression values) into a positive value, which will then increase rather than decrease the activity score *A_TG_*. As a result, *A_TG_* is a score designed to describe the regulatory activity of a TF through its target genes, rather than a score that measures the raw expression level of the target genes. A large *A_TG_* is an indication that the TF protein is actively modulating its target regulatory pathways and therefore functionally active. For example, if a TF acts mainly as a repressor in a cell type in which it is functionally active, we would observe low expression of its target genes, but high *A_TG_* because of the multiplier *s_g_* = -1 (**Figure S2A-B**). In contrast, for a TF that only acts as a positive regulator, high TG expression would equate to high *A_TG_*. In general, for TFs that can both activate and repress its target genes, a high *A_TG_* score means positively regulated target genes are highly expressed and negatively regulated target genes are lowly expressed, while a low *A_TG_* score means positively regulated target genes are lowly expressed and negatively regulated target genes are highly expressed.

In ChIP-PED, the target genes and their directionality, *s_g_*, are defined by users after they analyze their own ChIPx and gene expression data. It is possible that a gene is activated by a TF in one biological context, but is repressed by the TF in another context. Depending on the initial definition of the target gene as an activated or repressed target, the directionality, *s_g_*, may be opposite of the actual regulatory behavior in a specific context in the compendium. For instance, in the input target gene list, the directionality of the target gene may be specified as activated, *s_g_* = 1, but in truth, the target gene is repressed in a specific context A in the PED compendium. In this case, although the TF is functionally active in context A, the repressed target gene will suggest otherwise, since the target gene is defined as positively regulated by the user (i.e., *s_g_*=1) but its repressed expression will contribute a negative score to the total target gene activity, which will then reduce the total level of target gene activity (*A_TG_*) in that context. Thus, even though in the context, the TF may be strongly expressed, the reduced *A_TG_* due to repression rather than activation (or vice versa) of the defined target genes may cause the total TG activity to fall below the TG activity cutoff. As a result, the context may not be predicted as a TF+TG+ functionally active context and only the contexts enriched with regulatory activity consistent with the defined activated and repressed target gene input will be recovered. For this reason, contexts in which the TF may regulate different target genes or regulate them in a different manner (e.g. repress instead of activate) may not be found by ChIP-PED. This could be considered as a potential limitation of ChIP-PED and whether one can circumvent this limitation and how to circumvent is a topic worthy of future investigation.

The differences in activation and repression directions may also have implications on the definition of input target genes. When one has only one TF perturbation experiment for constructing the input target gene list, the direction of regulation (positive or negative) of each target gene will be easily defined. However, when one has multiple TF perturbation experiments, there will be many different ways to define target genes and their direction of regulation. In our analyses, when we use multiple ChIPx and TF perturbation data to construct input target genes for ChIP-PED analyses, we usually define target genes as genes that are differentially expressed in a majority of the experiments, and we only retain the target genes that are consistently activated (positive differential expression) or consistently repressed (negative differential expression). In other words, genes activated by the TF in one context, but repressed by the TF in another context are not used as our input target genes. In this way, our input target genes are likely to be core targets of the TF that behave consistently in different contexts. This could help users to identify TF regulatory activities from PED that are relatively more cell-type independent. Users, however, do have the flexibility to include cell type specific targets. When they do this, they have to decide which sign *s_g_* to use based on their specific needs, and remember that contexts with inconsistent regulation signs may be missed as explained in the previous paragraph.

## ChIP-PED regulatory patterns

ChIP-PED is capable of analyzing four regulatory patterns: TF+TG+, TF+TG-, TF-TG+, and TF-TG-. Biological contexts enriched with TF+TG+ samples are likely to be contexts in which the TF plays an active regulatory role through some or all of the input target genes since in these samples the TF is highly expressed AND the target gene activity is high. High TF expression alone is not sufficient to imply the existence of functional TF protein due to possible post-transcriptional and translational regulation, but high TG activity in addition to high TF expression would strongly support the presence of active TF protein. In the main article, we illustrated multiple examples of ChIP-PED TF+TG+ analyses and demonstrated how they can be used to obtain meaningful biological insights. Here, we will illustrate and discuss possible interpretations of ChIP-PED analyses using the other three regulatory patterns.

The TF-TG+ regulatory pattern searches for biological contexts in which the TF is lowly expressed and the TG activity is high. TF-TG+ may indicate the presence of other unknown positive regulators or failure to measure TF expression. For example, we used ChIP-PED to analyze the *MYC* data described in the main article for the TF-TG+ regulatory pattern. We found that Wilms tumors were significantly enriched with the TF-TG+ regulatory pattern (**Supplementary Table S3**). As can be seen in **Figure 2B**, Wilms tumors exhibit strong target gene activity even though *MYC* is lowly expressed. A possible reason for why *MYC* target genes are active in Wilms tumors even though *MYC* is lowly expressed is that another TF with a similar regulatory role to *MYC* is responsible. Alternatively, the experiments in Wilms tumor may simply have failed to accurately measure *MYC* expression.

The TF+TG- regulatory pattern searches for biological contexts in which the TF is highly expressed and the TG activity is low. TF+TG- may suggest non-functional TF protein, the existence of other negative regulators, or functional TF protein regulating a completely different set of target genes. For example, we used ChIP-PED to analyze the *Gata1* data described in the main article for the TF+TG- regulatory pattern. We found that hematopoietic stem cells were significantly enriched with the TF+TG- regulatory pattern (**Supplementary Table S4**). As can be seen in **Figure 2C**, the hematopoietic stem cells exhibit weak target gene activity, even though *Gata1* is highly expressed. A possible reason for this observation is that *Gata1* regulates slightly different sets of TF target genes during progressive stages of hematopoiesis, and thus the target genes constructed from later stages of hematopoietic cell development may not show heightened activity in hematopoietic stem cells. Alternatively, there may be a strong negative regulator that represses the expression of the target genes in hematopoietic stem cells prior to hematopoiesis.

The TF-TG- regulatory pattern searches for biological contexts in which the TF is lowly expressed and the TG activity is low. Since in almost all cases, users will be interested in searching for samples with regulatory activity of the TF, rather than searching for samples without regulatory activity of the TF, we do not describe an additional analysis example.

For the TF-TG+ and TF+TG- predictions from the *MYC* and *Gata1* analyses, we did not report supporting literature since it is difficult to pinpoint the exact functional mechanism or regulators due to the many possible explanations for the observed associations. It should be noted that even though regulatory patterns other than TF+TG+ may not be easily explained, the predicted biological contexts may still be interesting to researchers for further study. For example, researchers interested in the disease progression of Wilms tumor may want to further study why *MYC* target genes are active and its functional implications. We later illustrate how ChIP-PED can be used to quickly test if homologous TFs could be compensating for the low expression of *MYC* in Wilms tumors as a secondary regulator of the *MYC* target genes (**Supplementary Methods 1.7**).

## Function to perform ChIP-PED analysis

All ChIP-PED analyses performed in the main manuscript can be performed using the functions in the ChIP-PED R package available at [www.biostat.jhsph.edu/~gewu/ChIPPED](http://www.biostat.jhsph.edu/~gewu/ChIPPED). The main function in the package is ***ChIPPED***, which allows users to predict which contexts in either compendium of human or mouse PED are enriched with TF functional activity specified by a regulatory pattern of interest. In order to do so, ***ChIPPED*** requires the user to provide the Entrez Gene ID of the TF of interest, the Entrez Gene IDs of the activated and repressed target genes of the TF, a regulatory pattern of interest (e.g. TF+TG+), the species (e.g. Mouse), the TF and TG cutoff parameters, the Bonferroni adjusted p-value cutoff for significant enrichment, and whether further automated follow-up analyses should be performed.

As default, ***ChIPPED*** sets the TF and TG cutoff parameters to correspond to a one-sided p-value of 0.1 based on fitted normal distributions for the TF expression, *E_TF_*, and TG activity, *A_TG_*. These default parameters were used to perform all ChIP-PED analyses in the main manuscript.***­*** Users can change the default parameters to increase or decrease the TF and TG cutoff by specifying smaller or larger p-values, respectively. Users are also given the option to set the TF and TG cutoff parameters based on a desired quantile or a direct TF expression and TG activity value. The default Bonferroni adjusted p-value cutoff is 0.05, which will tell ***ChIPPED*** to report only the contexts with significant enrichment above the level corresponding to a Bonferroni adjusted p-value of 0.05. Users can modify this parameter to report more or less predictions depending on their desired significance level.

Users are also given an option to automatically perform follow-up ChIP-PED analyses after the initial ChIP-PED analysis. By default, ChIP-PED will not automatically perform follow-up ChIP-PED analyses. **Supplementary Methods 1.6-1.7** describe in full detail the exact follow-up ChIP-PED analyses performed, so we will not describe it in here.

Given the input, ***ChIPPED*** will search through the mouse or human compendium of publicly available gene expression data for biological contexts with enriched TF functional activity as specified by the regulatory pattern of interest and activated and repressed target genes. Specifically, ***ChIPPED*** will output a ranked table of biological contexts with (1) the number of samples in each context that exhibit the regulatory pattern of interest (Active), (2) the number of total samples for each predicted context (Total), (3) the fold change for each predicted context (FoldChange) which will be defined below, (4) the Bonferroni corrected p-value from the Fisher’s exact test of association between the context and the regulatory pattern of interest (Adj.Pvalue), (5) the label of the biological context (SampleType), (6) the experiments denoted by their GSE IDs that generated the samples of the context (Experiment), and (7) the regulatory pattern of interest (Pattern). FoldChange is calculated by:

(1)

where *N* is the total number of samples in the compendium, *K* is the total number of samples in the compendium that exhibit the regulatory pattern of interest, *n_C_* is the total number of samples in context *c*, and *k_C_* is the number of samples in context *c* that exhibit the regulatory pattern. The pseudocounts *K/N* and *1* are added to *k_C_* and *n_C_* to avoid unstable fold change estimates due to small sample counts. Numerous examples of the ranked table output of the ***ChIPPED*** function can be found in **Supplementary Tables S2-S7**.

## Functions to perform follow-up ChIP-PED analysis for more detailed exploration

After performing an initial ChIP-PED analysis, users may be interested in studying a few of the predicted TF-active biological contexts in more detail to obtain additional insight into the regulatory behavior of the TF in those specific biological contexts (e.g., compare the TF regulatory activity in a predicted context with the activities in other related but not necessarily enriched biological contexts in the compendium). The ChIP-PED package provides several functions to support such exploration.

First, the ChIP-PED package provides a function, ***tabSearch***, for users to search for available biological contexts of potential interest in our PED compendiums. To use this function, users input a species (i.e., human or mouse) and a combination of keywords such as cell type names (e.g., MCF7) and/or GEO experiment identifiers (e.g., GSE1234). The function will then search through the compendium and return a list of biological contexts whose biological context labels contain the specified keywords. For instance, if one uses “MCF7 AND GSE1234” as keyword, all biological contexts from the GEO series GSE1234 that contain MCF7 in the context labels will be returned.

Second, the ChIP-PED package provides another function, ***ChIPPEDeda***, to visualize and test for significant differences in mean TF expression, *E_TF_*, and mean TG activity, *A_TG_*, between user-specified biological contexts. To use this function, one must first specify a PED compendium, a TF of interest, a list of activated and repressed target genes of the TF, and a list of interesting biological contexts (e.g. the ***tabSearch*** results). ChIP-PED will then generate four plots:

1. A plot showing the mean ± one standard deviation (SD) of the *E_TF_* and *A_TG_* values for each biological context of interest (e.g., **Figure S6A**)**.** The sample count for each context will be shown in parenthesis next to each biological context label. Contexts with only one sample will have SD=0, so they will have a dot rather than lines representing ±1 SD. When creating this plot, users also have the option to order the biological contexts based on the mean *E_TF_* (high to low), the mean *A_TF_* (high to low), or the average rank of *E_TF_* and *A_TG_* (i.e., first rank the user-specified contexts by mean *E_TF_* and mean *A_TG_* respectively, then compute the average of the two ranks of each context, and finally re-order the contexts based on the average rank).
2. A ChIP-PED plot of *A_TG_* versus *E_TF_* (e.g. **Figure S6B**) values for all samples in the compendium, where samples from the user-specified biological contexts are highlighted in different colors.
   1. Two heatmaps depicting the t-statistics and p-values from t-tests comparing the mean *E_TF_* and mean *A_TG_* values of all pair-wise combinations of the user-specified contexts (e.g. **Figure S6C-D**). The top half of the each heatmap will contain the *A_TG_* results and the bottom half of each heatmap will contain the *E_TF_* results. Contexts are ordered from top to bottom (rows) and right to left (columns) according to the ranking order specified by the user (i.e. mean *E_TF_*, mean *A_TG_*, or average rank of *E_TF_* and *A_TG_*). All mean differences calculated in the t-tests are calculated by subtracting the lower ranked contexts from the higher ranked contexts. P-values will also be Bonferroni corrected based on the number of pair-wise t-tests performed.

In addition, the usual ChIP-PED results will also be reported: for each input context, the proportion of samples that exhibit the regulatory pattern of interest (e.g., TF+TG+) will be calculated, and Fisher’s exact tests will be performed to test the association of the context with the regulatory pattern. The ChIP-PED analysis results with all data and summary tables, including the raw *E_TF_* and *A_TG_* values for each user-specified context, used to generate the plots will be exported to csv files in a directory specified by the user (e.g., worksheet named “STAT1 ChIPPEDeda Results” in **Supplementary Table S5**). Since the exported data also contains the raw *E_TF_* and *A_TG_* values, the users can easily perform any additional follow-up statistical analyses, especially given the wealth of available statistical functions and packages for R.

## Summary of ChIP-PED analyses

**Supplementary Table S12** summarizes the functions available in the ChIP-PED package. Users can use these functions in three suggested modes: fully automated ChIP-PED analysis, automated ChIP-PED analysis with manual follow-up ChIP-PED analyses, and manual ChIP-PED exploration.

If users want to perform a fully automated ChIP-PED analysis, users need only to run the ***ChIPPED*** function with the option to perform automated follow-up ChIP-PED analyses set to TRUE. ChIP-PED will first perform an initial ChIP-PED analysis. Then ChIP-PED will extract the GEO experiment ID (i.e. GSE***) for each predicted biological context and use ***tabSearch*** to identify all biological contexts in each experiment. Next, ***ChIPPEDeda*** will iteratively analyze each set of identified biological contexts from each experiment. The reason for using the experiment ID to search for related biological contexts is because it is common for experiments to be conducted to study the similarities and differences among related biological contexts, thus providing a quick and easy source for interesting contexts for comparison. A specific example is provided in **Supplementary Method 1.11**.

Alternatively, users can first perform an initial ChIP-PED analysis and then manually perform follow-up ChIP-PED analyses. For instance, if the initial ChIP-PED analysis predicts “fetal liver” as an enriched context, the user may use “liver” as the keyword to search for other biological contexts with “liver” in the context annotations, and then use ***ChIPPEDeda*** to generate plots similar to **Figure S6** to explore the TF regulatory activities in the recovered liver-related contexts. This would then result in a different analysis than the fully automated analysis, since “liver” is much more general (e.g., will include many more contexts from multiple experiments) than a single experiment ID.

Also, users can directly skip the initial ChIP-PED analysis and perform manual ChIP-PED exploration using ***tabSearch*** and ***ChIPPEDeda*** to search for TF regulatory activity in any list of contexts that are interesting to the user. For example, in the *MYC* TF-TG+ ChIP-PED analysis, we found that *MYC* was not highly expressed in Wilms tumors, even though the *MYC* target genes were highly expressed (**Figures 2B, S10**). One possible reason for this observation was that homologous TFs, such as *MYCN* and *MYCL* in the MYC family, were activating a subset of the *MYC* target genes in place of *MYC*. To quickly explore this possibility without first performing initial ChIP-PED analyses for *MYCN* and *MYCL*, we used ***tabSearch*** to search for all Wilms tumor related contexts using “Wilms tumor” as a keyword and then used ***ChIPPEDeda*** to test if any of the recovered “Wilms tumor” contexts exhibited high *MYCN* or *MYCL* expression and high *MYC* target gene activity **(Supplementary Table S3)**. Unfortunately, we did not find statistically significant enrichment of TF+TG+ functional activity for *MYCN* or for *MYCL* **(Supplementary Table S3)**. When we visualized how the regulatory pattern changed when *MYCN* or *MYCL* expression is shown in place of *MYC* expression in the ChIP-PED plots generated by ***ChIPPEDeda*** (**Figure S10**), we found that *MYCL* was lowly expressed in Wilms tumor samples, but *MYCN* was highly expressed in Wilms tumor samples. Thus, the lack of significance for *MYCN* even though *MYCN* is highly expressed, is primarily because the proportion of samples with *A_TG_* score above the TG+ cutoff is not large enough compared to the proportion of background TF+TG+ samples, which suggests that we may not have enough power to detect significant *MYCN* functional activity through *MYC* target genes. Despite this, the Wilms tumor samples that fall within the TF-TG+ region in the *MYC* plot were also found in the TF+TG+ region in the *MYCN* plot (**Figure S10**), therefore *MYCN* may still be a candidate that could compensate for *MYC* regulatory activity in Wilms tumor. In contrast, *MYCL* has a low *E_TF_* score, thus it is less likely that *MYCL* compensates for *MYC* regulatory activity in Wilms tumor.

Although our analysis of homologous TFs was inconclusive in this particular example, it demonstrates how users can flexibly use the ChIP-PED functions to manually study different hypotheses of interest in the future. In principle, rather than checking for homologous TF activity only in contexts enriched with TF-TG+ samples, one could also use this function to study homologous TF regulatory activity in contexts enriched with TF+TG+ regulatory activity. This would then help researchers understand whether only the TF of interest is activating and repressing its TF target genes or possibly other homologous TFs with overlapping regulatory functions are also influencing TF target gene activity. Together these analyses using ***ChIPPEDeda*** would paint a clearer picture of the underlying regulatory network in the contexts of interest. More work in the future will still be needed to define a more rigorous and effective method to jointly examine functional activity of homologous or cooperative TFs along with the TF of interest in different biological contexts.

## MYC experiment protocol

To validate the novel ChIP-PED prediction that *MYC* was functionally active in Ewing sarcoma, we performed the following experiments:

*Western Blot:* Whole cell lysates were extracted using RIPA lysis buffer from a Ewing sarcoma cell line TC71 stably expressing control vector or shMyc/pLKO.1 vector. Protein lysates were quantified using Pierce BSA protein assay. 40 micrograms/well were loaded on a 10% SDS-PAGE gel and subsequently transferred to PVDF membrane. Membrane was blocked with 5% Milk/TBST solution and then exposed to rabbit anti-Myc primary antibody (Epitomics, Inc.) overnight at 4 degrees Celsius. Membrane was washed x 3 with TBST and then exposed to HRP-conjugated goat anti-rabbit secondary antibody (Santa Cruz Laboratories) for 1hour a room temperature. After washing with TBST x3, blot was exposed with chemilluminescence. Western blots are performed and developed separately under different exposure times, thus intensity differences between pre and post 6-week blots are likely to be due to blot film exposure. Even so, we cannot rule out the possibility that the *in vivo* microenvironment of the xenograft tumors may enhance c-Myc expression compared to *in vitro* culture conditions. More importantly, both western blots in **Figure 4E** accurately depict the differential protein expression observed between the control and shMyc cells for both the *in vivo* and *in vitro* studies.

*Soft Agar Assay:* 5,000 TC71 control and shMyc cells were placed into 0.4% agarose that was subsequently placed onto a basement 0.8% agarose layer in a 6 well plate. After solidification, 1 ml of full media was placed onto the top layer. Wells were monitored for 2-3 weeks for colony formation. Triplicate wells were performed.

*Xenograft experiments:* 1 million TC71 control and shMyc cells were subcutaneously injected into the dorsal flank of five NOD/SCID/IL-2γ null (NSG) mice for each cell type and monitored for tumor development. After 6 weeks, animals were sacrificed per institutional IACUC protocol and primary tumors were weighed. Experiment was repeated with similar results noted.

*Proliferation assay:* 2,000 TC71 control and shMyc cells in 200 microliters of full media were initially placed into duplicate wells and monitored via use of CCK-8 (Dojindo, Inc.) cell proliferation and viability assay per manufacturer’s protocol. Briefly, 10 microliters of CCK-8 solution was added daily into duplicate wells and allowed to incubate for 4 hours at standard tissue culture conditions (37ºC). Subsequently the 96 well plate was placed into a microplate reader and absorbance measured at 450nm. Duplicate absorbance measurements were recorded and averaged for each condition and time point. Similar protocol was followed for MHH-ES control and shMyc cells.

## Biological information in gene expression compendium can be meaningfully captured in spite of data heterogeneity

ChIP-PED integrates ChIPx (ChIP-chip or ChIP-seq) data with a large compendium of publicly available gene expression data to expand the scope of possible discoveries made from a ChIPx experiment. Since the compiled data is from many different cell types, labs, and experiments, a major concern is whether the lab or batch effects inherent in the highly heterogeneous data may overwhelm the biological signal. To check whether this was true in the GPL96 and GPL1261 compendiums, we calculated the Euclidean distance between three groups of samples: samples from the same tissues and the same lab, samples from different tissues and the same lab, and samples from the same tissues but different labs (**Figure S4A**). We found that the distance between samples from the same tissue and the same lab was smallest. More importantly, we found that samples from the same tissue and different labs were closer in similarity than different tissues from the same lab.

As a further demonstration that the biological similarity in tissues was stronger than the possible lab or batch effects, we hierarchically clustered a few tissues – liver, kidney, heart, brain, and lung – that were measured by multiple labs and display the results in a heatmap (**Figure S4B**). We found that samples of the same tissue type clustered together despite originating from different labs and experiments. Our results support similar findings by others (Zilliox *et al.*, 2007; McCall *et al.*, 2011) that the lab or batch effects are not significant enough to overwhelm the biological signal in the large compendiums of gene expression data.

## ChIP-PED can predict functional biological contexts in spite of the potential cell type specificity of target genes

Conceptually, ChIP-PED uses target genes obtained from one biological context to predict TF regulatory activity in other biological contexts. If the two biological contexts do not share any target genes, then it would not be possible to predict either contexts using the target genes defined from the other context. However, a number of recent studies have shown that many TFs do have a core set of target genes that are either cell type independent or shared by many cell types in which the TF is functionally active (Lee *et al*., 2010; Ji *et al.*, 2011). Therefore, it is not uncommon that contexts share at least some target genes. The shared targets provide the foundation for discovering new (but not necessarily all) biological contexts associated with regulatory activity of the TF.

To test whether ChIP-PED can remain effective when the majority of target genes are cell-type specific, we examined *Stat3* in mouse using matching ChIPx and gene perturbation data in Th17 cells and CD4+ T cells (**Supplementary Table S1, S8**). Th17 cells and CD4+ T cells are two distinct cell types. Naïve CD4+ T cells when appropriately activated can differentiate into Th17 cells. *Stat3* has been shown to be critically important in the regulation of each cell type through distinct target genes (Durant *et al.* 2010; Kwon *et al.* 2009), thus providing us with an opportunity to compare the regulatory activity of a TF in cell types in which the TF has been proven to be functionally active. Target genes were constructed by intersecting differentially expressed genes with TF-bound genes as described previously.

We found that even though only 33 out of 313 (10.5%) Th17 *Stat3* target genes overlapped with CD4+ T cell target genes, ChIP-PED was able to identify CD4+ T cells as enriched TF+TG+ biological contexts using *Stat3* target genes in Th17 cells (**Figure S5, Supplementary Table S8**). Similarly, *Stat3* regulatory activity computed using targets in CD4+ T cells was significantly enriched in Th17 cells, even though only 33 out of 127 (26.0%) of the CD4+ T cell *Stat3* target genes overlapped with Th17 *Stat3* target genes (**Figure S5, Supplementary Table S8**). This shows that in spite of the cell-type specificity of most of the TF target genes, as long as there exists a small, but highly active set of overlapping TF target genes, as seen for *Stat3* in Th17 and CD4+ T cells, it is possible to predict other cell types that share similar TF regulatory activity.

As a reminder, not all biological contexts known to be enriched with regulatory activity of a TF will be recovered by ChIP-PED. This is because there may be no target gene overlap between the contexts(s) used to define the target genes and some of the other active contexts. These situations may arise, for example, if the TF plays a completely different functional role through a separate pathway – i.e., a completely unique set of activated and repressed target genes. Even so, this does not mean ChIP-PED is not useful, since it is still able to recover many biological contexts with shared target genes and functional activity, which, as we have demonstrated in the main article, may lead to many known and novel functional connections.

## Follow-up ChIP-PED analysis of STAT1 functional activity in hepatitis C infected PBMCs

As discussed in the main manuscript and in **Supplementary Method 1.6-1.7,** ChIP-PED can automatically perform follow-up analyses to study each predicted biological context of interest in more detail. We used this procedure after the initial *STAT1* ChIP-PED analysis described in the main manuscript to further study the regulatory behavior of *STAT1* in predicted *STAT1*-active PBMCs infected with hepatitis C from GSE7123. ***tabSearch*** found 6 biological contexts in GSE7123: PBMCs from healthy patients and PBMCs from hepatitis C infected patients given treatment with interferon and ribavirin and then allowed to recover for 1, 2, 7, 14, and 28 days. After the follow-up analysis of the contexts in GSE7123 using ***ChIPPEDeda***, we found that healthy PBMCs had lower *STAT1* expression, *E_TF_*, and TG activity, *A_TG_*, than infected PBMCs, and as the recovery days increased, *STAT1* functional activity decreased as measured by both *E_TF_* and *A_TG_* (**Figure S6A-B**). This was further reinforced by the pairwise t-tests between each of the contexts, which showed that healthy PBMCs had significantly lower *E_TF_* and *A_TG_* mean values than infected PBMCs, and infected PBMCs from day 7, 14, and 28 had significantly lower *E_TF_* and *A_TG_* mean values than infected PBMCs from day 1 and 2 (**Figure S6C-D**). The initial ChIP-PED results also reflected this trend as seen by the decreasing proportion of STAT1+TG+ contexts from day 1 to day 28 infected PBMCs and the enrichment of STAT1+TG+ functional activity only in infected PBMCs compared to healthy PBMCs (**Supplementary Table S5**). None of these findings were reported by either of the two publications that generated the STAT1 ChIPx data (Robertson *et al.*, 2007) or the PBMCs gene expression data from GSE7123 (Taylor *et al.*, 2007). Thus, by performing follow-up ChIPPED analyses, users may be able to make functional discoveries that they otherwise would have not been able to when using standard ChIPx analyses.

## Effect of different methods to construct target genes on ChIP-PED analyses

In our main manuscript, we choose to define a gene as TF-bound if a significant peak detected from ChIPx data overlapped with the -10kb to +5kb region around the TSS of a gene. Many TFs regulate their target genes by binding to distal regulatory elements such as enhancers (Ernst *et al*., 2011; McLean *et al.,* 2010). Thus a potential concern of using the -10kb to +5kb region around the TSS is it may miss target genes regulated through distal TFBSs further than through TFBSs located within the annotation window region. To explore the effectiveness of our default annotation window size on ChIP-PED analyses, we compared ChIP-PED prediction performance using two additional methods to construct target genes, which are designed to capture more distal regulatory targets. First, we compared ChIP-PED predictions using target genes defined from ChIP-seq and TF perturbation data to ChIP-PED predictions using target genes empirically defined from ChIA-PET and TF perturbation data. Second, we compared ChIP-PED results by using different annotation window sizes to construct TF target genes.

ChIA-PET, chromatin interaction analysis by paired-end tag sequencing, is a high-throughput approach to map TFBSs and their interacting *cis*-regulatory elements in the genome. Besides providing the locations of the TFBSs and their interacting elements, ChIA-PET also provides information on pairwise interactions among the mapped locations. This allows ChIA-PET to be able to detect long-range chromatin interactions, including interactions between distal enhancers and proximal promoters (Fullwood *et al.*, 2009). Thus, researchers may be able to more accurately annotate target genes regulated by distal regulatory elements using ChIA-PET. Compared to ChIA-PET, ChIP-seq is also capable of identifying both proximal and distal TFBSs. However, ChIP-seq does not provide information on how different *cis*-regulatory elements interact, and as a result, one cannot tell from the ChIP-seq data whether a distal TFBS directly regulates a particular gene as opposed to regulating other genes. Since the ChIA-PET method allows one to better annotate TF bound genes to distal regulatory elements, the target genes constructed from the TF-bound genes detected by ChIA-PET may be able to more accurately define distal regulated target genes. By comparing ChIP-PED analyses using target gene constructed from ChIA-PET to ChIP-PED analyses using target genes constructed from ChIP-seq, the results may shed light on how different target gene construction methods affect prediction performance.

Currently, there are not many published ChIA-PET datasets. Fortunately, for *ESR1*, both ChIA-PET and ChIP-seq data are available in the MCF7 cell line. Unfortunately, for the TFs other than *ESR1*, no matching ChIA-PET data are available. We therefore used ChIP-PED to analyze *ESR1* target genes defined using both ChIP-seq and ChIA-PET data from MCF7 cells treated with estrogen and gene expression data from MCF7 cells before and after treatment with estrogen (**Supplementary Table S7**). ESR1-bound genes defined by ChIA-PET were extracted directly from the original ChIA-PET publication (Fullwood *et al*., 2009) by intersecting *ESR1*-bound genes from datasets IHH015F and IHM001F. *ESR1*-bound genes defined by ChIP-seq were obtained by detecting significant peaks in GSE14664 using CisGenome at a FDR ≤ 0.1 with the default annotation window size of -10kbp to +5kbp around the gene TSS. Then both sets of ESR1-bound genes were intersected with differentially expressed genes obtained using RMA and limma to analyze the gene expression data from MCF7 cells before and after treatment with estrogen in GSE11791. In total, 735 *ESR1*-bound genes were shared between 1653 (44.5%) ChIA-PET derived *ESR1*-bound genes and 1635 (45.0%) ChIP-seq derived *ESR1*-bound genes. After intersection with the gene expression data, 142 (105 activated and 37 repressed) *ESR1* target genes were shared between 278 (51.1%) ChIA-PET derived *ESR1*-bound genes and 247 (57.5%) ChIP-seq derived *ESR1*-bound genes.

We then analyzed both sets of target genes using ChIP-PED and found that most predictions were identical between the two methods and ChIP-PED prediction accuracy was slightly higher using ChIA-PET rather than ChIP-seq to define target genes (**Supplementary Table S7**). In total, 22 out of the 23 ChIA-PET derived predictions were identical to a ChIP-seq derived prediction. 43.5% (10/23) of the predictions were functionally validated and 95.7% (22/23) of the predictions were functionally validated or indirectly supported in literature. In comparison, 22 out of the 27 ChIP-seq derived predictions were identical to a ChIA-PET derived prediction. 40.7% (11/27) of the predictions were functionally validated and 92.6% (25/27) of the predictions were functionally validated or indirectly supported in literature. Altogether, these results suggest that either using ChIA-PET to derive target genes or ChIP-seq will both result in comparable ChIP-PED predictions. Although we did not find a large difference between ChIA-PET and ChIP-PED predictions in our analysis, which uses ChIA-PET data generated for *ESR1* from one of the first ChIA-PET experiments, it is possible that the ChIP-PED analyses based on future ChIA-PET analyses may have improved performance due to maturation of ChIA-PET technology and better experimental protocols.

We further explored different annotation window sizes in order to evaluate the effect of annotation window size on ChIP-PED performance. In total, we tested 7 different annotation window sizes around the gene TSS: -5kbp to +2.5kbp, -10kbp to +5kbp (default), -25kbp to +12.5kbp, -50kbp to +25kbp, -100kbp to +50kbp, -1000kbp to +500kbp, and an infinite annotation window size (which equates to directly defining all differentially expressed genes in the TF perturbation data as target genes, rather than first intersecting with ChIP-seq TF-bound genes). We performed ChIP-PED analyses on 5 TFs: *Oct4, Gata1, Jarid2, MYC,* and *ESR1*, using each annotation window size to define target genes. We then tabulated the total number of predictions that were directly supported by functional evidence or indirectly supported by existing literature as described in the main article (**Supplementary Table S9**). *STAT1* is excluded in this analysis because all annotation window sizes resulted in the same 23 target genes and the same ChIP-PED predictions. This is because only in the *STAT1* analysis was target genes required to both TF-bound and strictly validated by functional experiments in literature (rather than TF perturbation data). We found that overall prediction accuracy across the different windows sizes was very similar: functionally supported prediction accuracy was 48.4%, 50.6%, 50.7%, 51.2%, 50.0%, 50.8%, and 48.8%, and functionally or indirectly supported prediction accuracy was 79.1%, 78.3%, 79.6%, 81.6%, 80.6%, 81.4%, and 80.2% for -5kbp to +2.5kbp, -10kbp to +5kbp, -25kbp to +12.5kbp, -50kbp to +25kbp, -100kbp to +50kbp, -1000kbp to +500kbp, and infinite window size, respectively (**Supplementary Table S10**). Even though prediction accuracies were relatively similar, the number of functional and/or indirectly supported predictions for the -10kbp to +5kbp annotation window size was the highest (84 functional and 130 functional or indirectly supported predictions) compared to the other window sizes, which ranged from 59 to 77 functional and 96 to 121 functional or indirectly supported predictions (**Supplementary Table S10**). Thus, our data suggests that the -10kbp to +5kbp annotation window is the best performing overall since it is able to recover the largest number of functional and/or indirectly supported predictions (i.e. higher power) without sacrificing much prediction accuracy.

## Median TF expression and TG activity measure

We also investigated how using the median to measure TF expression and TG activity, instead of the default mean expression and activity, would impact ChIP-PED prediction performance. Specifically, TF expression, *E_TF_*, was modified to be the median of *p_TF_,* the normalized probeset intensities for each TF:

(2)

. Similarly, the sign-adjusted expression of target gene *g* was:

(3)

where *p_g_* is the set of normalized probeset intensities of each target gene *g* and *s_g_* is a multiplier of 1 or -1 depending on whether gene *g* is positively regulated (activated) or negatively regulated (repressed), respectively. Finally, target gene activity, *A_TG_*, was then defined as the median of the expression of all target genes:

(4)

In contrast, the original ChIP-PED method uses the mean, rather than median, probeset intensities to calculate the expression of each TF and each target gene (for 2-3). Then, the original ChIP-PED method uses the mean, rather than median, expression of all target genes to calculate target gene activity (for 4).

We used these two modified median measures of TF expression and TG activity to repeat the ChIP-PED analyses for each of the six TFs. We found that correlation between *E_TF_* and *A_TG_* values for each of the TFs and the predicted biological contexts from both measures did not substantially differ (**Figure S9**, **Supplementary Table 2-7)**. In total, 98.8% (171/173) of the median based predictions were identical to a mean based prediction and 96.1% (171/178) of the mean based predictions were identical to a median based prediction (**Supplementary Table S2-S7)**. The prediction accuracy of the median and mean measure was also approximately the same: for the median measure, 88/173 (50.9%) predictions were functionally validated and 136/173 (78.6%) predictions were functionally validated or indirectly supported in literature compared to 90/178 (50.6%) functionally validated predictions and 141/178 (79.2%) functionally validated or indirectly supported predictions for the mean measure (**Supplementary Table S2-S7)**. Thus, we find that ChIP-PED analyses based on the median TF and TG measure are very similar to ChIP-PED analyses based on the mean TF and TG measure in terms of ChIP-PED prediction accuracy and the predicted TF-active contexts.

## Effect of biological context sample count on prediction accuracy

Since different biological contexts have different numbers of samples in the compendiums (**Supplementary Figure S1**), a natural question is whether ChIP-PED prediction accuracy is correlated with the number of samples in the compendium for the predicted context. To investigate this, we tabulated the predictions made in the ChIP-PED analyses of *Oct4, Jarid2, Gata1, MYC, ESR1,* and *STAT1*, to see if there was a clear association between prediction accuracy and biological context sample count (**Supplementary Table S2-S7**). Although one may speculate that the prediction accuracy may monotonically increase with the number of samples in a biological context, such a trend was not observed (**Supplementary** **Table S11)**. In fact, the prediction accuracy when the context sample count is between 4 and 5 was lower than the accuracy when the context sample count equal to 3 or between 6 and 15. Therefore there does not seem to be a simple monotone relationship between the prediction accuracy and context sample count.

## Selection of TF and TG cutoffs

By default, the TF and TG cutoff is chosen to be values corresponding to a one-sided p-value of 0.1 based on fitted normal distributions for *E_TF_* or *A_TG_* across all samples. This cutoff can be adjusted depending on factors such as how much resources are available to the investigator to experimentally validate novel biological contexts predicted by ChIP-PED or the user’s definition of what activity or expression levels are considered high enough to be biologically interesting. For example, in the *Oct4* analysis, if one choses a more stringent cutoff corresponding to a one-sided p-value of 0.01 to define high TF and high TG activity, 91.3% (21/23) of TF+TG+ contexts would be +*Oct4* biological contexts, and only 8.6% (2/23) would be -*Oct4* samples, representing a lower false discovery rate (compared to 3/28 = 10.7% for a TF/TG cutoff corresponding to a one-sided p-value of 0.1). Thus, a user can decide to decrease the false discovery rate by making the TF and TG cutoffs more stringent, if they prefer to obtain a smaller number of predictions that together are more likely to be true.

References

Anders,S. and Huber,W. (2010) Differential expression analysis for sequence count data. *Genome Biol.* **11**, R106.

Barrett,T. *et al.* (2009) NCBI GEO: archive for high-throughput functional genomic data. *Nucleic Acids Res.*, **37**, D885-890.

Chen L. *et al.* (2011) hmChIP: a database and web server for exploring publicly available human and mouse ChIP-seq and ChIP-chip data. *Bioinformatics* **27**, 1447-1448.

Durant,L. *et al.* (2010) Diverse targets of the transcription factor STAT3 contribute to T cell pathogenicity and homeostasis. *Immunity*, **32**, 605-615.

ENCODE Project Consortium *et al.* (2011) A user’s guide to the encyclopedia of DNA elements (ENCODE). *PLoS Biol.*, **9**, e1001046.

Ernst,J. *et al.* (2011) Mapping and analysis of chromatin state dynamics in the nine human cell types. *Nature* **473**, 43-49.

Johnson,E. *et al.* (2006) Model-based analysis of tiling-arrays for ChIP-chip. *Proc. Natl. Acad. Sci.* **103**, 12457-12462.

Fullwood, M. *et al.* (2009) An oestrogen-recepter-α-bound human chromatin interactome. *Nature* **462**, 58-64.

Irizarry,R. *et al.* (2003) Exploration, normalization, and summaries of high density oligonucleotide array probe level data. *Biostatistics* **4**, 249-264.

Ji,H. *et al.* (2008) An integrated system CisGenome for analyzing ChIP-chip and ChIP-seq data. *Nat. Biotechnol.*, **11**, 1293.

Ji,H. *et al.* (2011) Cell-type independent MYC target genes reveal a primordial signature involved in biomass accumulation. *PLoS One*, **6**, e26057.

Ji,H. *et al.* (2005) TileMap: create chromosomal map of tiling array hybridizations. *Bioinformatics* **21**, 3629-3636.

Kapur,K. *et al.* (2008) Cross-hybridization modeling on Affymetrix exon arrays. *Bioinformatics* **24**, 2887-2893.

Kwon,H. *et al.* (2009) Analysis of interleukin-21-induced Prdm1 gene regulation reveals functional cooperation of STAT3 and IRF4 transcription factors. *Immunity*, **18**, 941-952.

Lachmann,A. *et al.* (2010) ChEA: transcription factor regulation inferred from integrating genome-wide ChIP-X experiments. *Bioinformatics* **26**, 2438-2444.

Lee,E. *et al.* (2010) Hedgehog pathway-regulated gene networks in cerebellum development and tumorigenesis. *Proc. Natl. Acad. Sci. USA.* **107**, 9736-9741.

Liu,T. *et al.* (2011) Cistrome: an integrated platform for transcriptional regulation studies. *Genome Biol.* **12**, R83.

McCall,M.N. *et al.* (2010) Frozen robust multiarray analysis (fRMA). *Biostatistics*, **11**, 242-253

McCall,M. *et al.* (2011) The Gene Expression Barcode: leveraging public data repositories to begin cataloging the human and marine transcriptomes. *Nucleic Acid Res*. **39**, D1011-1015.

McLean,C.. *et al.* (2010) GREAT improves functional interpretation of *cis*-regulatory regions. *Nat. Biotechnol.* **28**, 495-501.

Robertson,G. *et al.* (2007) Genome-wide profiles of STAT1 DNA association using chromatin immunoprecipitation and massively parallel sequencing. *Nat. Methods*, **4**, 651-657.

Robinson,M. *et al.* (2010) edgeR: a Bioconductor package for differential expression analysis of digital gene expression data. *Bioinformatics* **26**, 139-140.

Smyth,G. (2004) Linear models and empirical Bayes methods for assessing differential expression in microarray experiments. *Stat. Appl. Genet. Mol. Biol.* **3**, Article 3.

Taylor,M. *et al.* (2007) Changes in gene expression during pegylated interferon and ribavirin therapy of chronic hepatitis C virus distinguish responders from nonresponders to antiviral therapy. *J. Virol.*, **81**, 3391-3401.

Trapnell,C. *et al.* (2013) Differential analysis of gene regulation at transcript resolution with RNA-seq*. Nat. Biotech.* **31**, 46-53.

Whiteld,T. *et al.* (2012) Functional analysis of transcription factor binding sites in human promoters. *Genome Biol.* **13**, R50.

Zhang,Y. *et al.* (2008) Model-based analysis of ChIP-seq (MACS). *Genome Biol.* **9**, R137.

Zilliox M. and Irizarry R. (2007) A gene expression bar code for microarray data. *Nat. Methods.* **4**, 911-913.

**Table S1.**ChIP-PED experimental test data

| TF | Cell Type | Species | Type | Source | Genes± |
| --- | --- | --- | --- | --- | --- |
| ESR1 | MCF7-E2 | Human | CS | GSE14664 | 1635 |
| ESR1 | MCF7-E2 | Human | TFP | GSE11791 | 1892 |
| Gata1 | G1E | Mouse | CS | GSE18164 | 3867 |
| Gata1 | G1ME | Mouse | TFP | GSE14980 | 2061 |
| Jarid2 | ESC | Mouse | CS | GSE18776 | 1053 |
| Jarid2 | ESC | Mouse | TFP | GSE19165 | 3547 |
| MYC | GM12878 | Human | CS | Encode-Uta | 5992 |
| MYC | H1HESC | Human | CS | Encode-Uta | 1716 |
| MYC | HELAS3 | Human | CS | Encode-Uta | 3983 |
| MYC | HEPG2 | Human | CS | Encode-Uta | 9703 |
| MYC | HUVEC | Human | CS | Encode-Uta | 9914 |
| MYC | K562 | Human | CS | Encode-Uta | 9080 |
| MYC | MCF7 | Human | CS | Encode-Uta | 1191 |
| MYC | HELAS3 | Human | CS | Encode-Yale | 8135 |
| MYC | K562 | Human | CS | Encode-Yale | 7320 |
| MYC | K562-Ifna30m | Human | CS | Encode-Yale | 5550 |
| MYC | K562-Ifna6h | Human | CS | Encode-Yale | 7186 |
| MYC | K562-ifng6h | Human | CS | Encode-Yale | 8194 |
| MYC | BT474 | Human | TFP | GSE5823 | 807 |
| MYC | HELAS3 | Human | TFP | GSE5823 | 3026 |
| MYC | MDA-MB231 | Human | TFP | GSE5823 | 1404 |
| MYC | MCF7 | Human | TFP | GSE5823 | 6508 |
| MYC | MCF7 | Human | TFP | GSE11791 | 859 |
| MYC | P493 | Human | TFP | GSE14302 | 10191 |
| MYC | P493 | Human | TFP | GSE14302 | 10664 |
| MYC | P493 | Human | TFP | GSE19703 | 6637 |
| Oct4 | ESC | Mouse | CS | GSE11431 | 1353 |
| Oct4 | ESC | Mouse | CS | GSE4189 | 9689 |
| STAT1 | HELAS3 | Human | CS+L | GSE15353 | 23 |
| Stat3 | CD4+T | Mouse | CS | GSE19198 | 1089 |
| Stat3 | CD4+T | Mouse | TFP | GSE19198 | 339 |
| Stat3 | Th17 | Mouse | CS | GSE21671 | 2531 |
| Stat3 | Th17 | Mouse | TFP | GSE21671 | 788 |

CS – ChIP-seq; TFP – TF perturbation; CS+L – ChIP-seq+literature

± If type is CS, then Genes is # of TF-bound genes and if type is TFP, then Genes is # of differentially expressed genes

**Table S10.**Prediction accuracy of different annotation window sizes for defining TF-bound genes

| Window (kbp) | Functional | Functional or Suggested |
| --- | --- | --- |
| -5 to +2. | 48.4% (**74**/153) | 79.1% (**121**/153) |
| -10 to +5 | 50.6% (**84**/166) | 78.3% (**130**/166) |
| -25 to +12.5 | 50.7% (**77**/152) | 79.6% (**121**/152) |
| -50 to +25 | 51.2% (**64**/125) | 81.6% (**102**/125) |
| -100 to +50 | 50.0% (**62**/124) | 80.6% (**100**/124) |
| -1000 to +500 | 50.8% (**60**/118) | 81.4% (**96**/118) |
| -∞ to +∞ | 48.8% (**59**/121) | 80.2% (**97**/121) |

*Annotation window sizes correspond to the region upstream (-) and downstream (+) of the TSS of each gene

**Table S11.**Biological context count prediction accuracy

| Context Count* | Functional | Functional or Suggested |
| --- | --- | --- |
| 16+ | 59.3% (16/27) | 88.9% (24/27) |
| 6 to 15 | 61.0% (25/41) | 82.9% (34/41) |
| 4 to 5 | 36.7% (18/49) | 73.5% (36/49) |
| 3 | 50.8% (31/61) | 77.0% (47/61) |

*Context count refers to the number of samples in the compendium for a given context

**Table S12.**ChIPPED R package functions

| Function | Purpose |
| --- | --- |
| ChIPPED | Identify biological contexts enriched with the TF regulatory activity given the TF target genes and regulatory pattern of interest. |
| tabSearch | Search for related contexts matching user-specified keywords. |
| ChIPPEDeda | Further explore initial ChIP-PED analyses by visualizing and testing for significant differences in mean TF expression and mean TG activity across a set of related contexts. |
| ConstructTG | Derive activated and repressed target genes of a TF given TF-bound genes from ChIPx data and differentially expressed genes from TF perturbation data. |
| annotatePeaks | Annotate each TF binding site found in ChIPx data with the corresponding regulatory targets in a given annotation window region. |

**
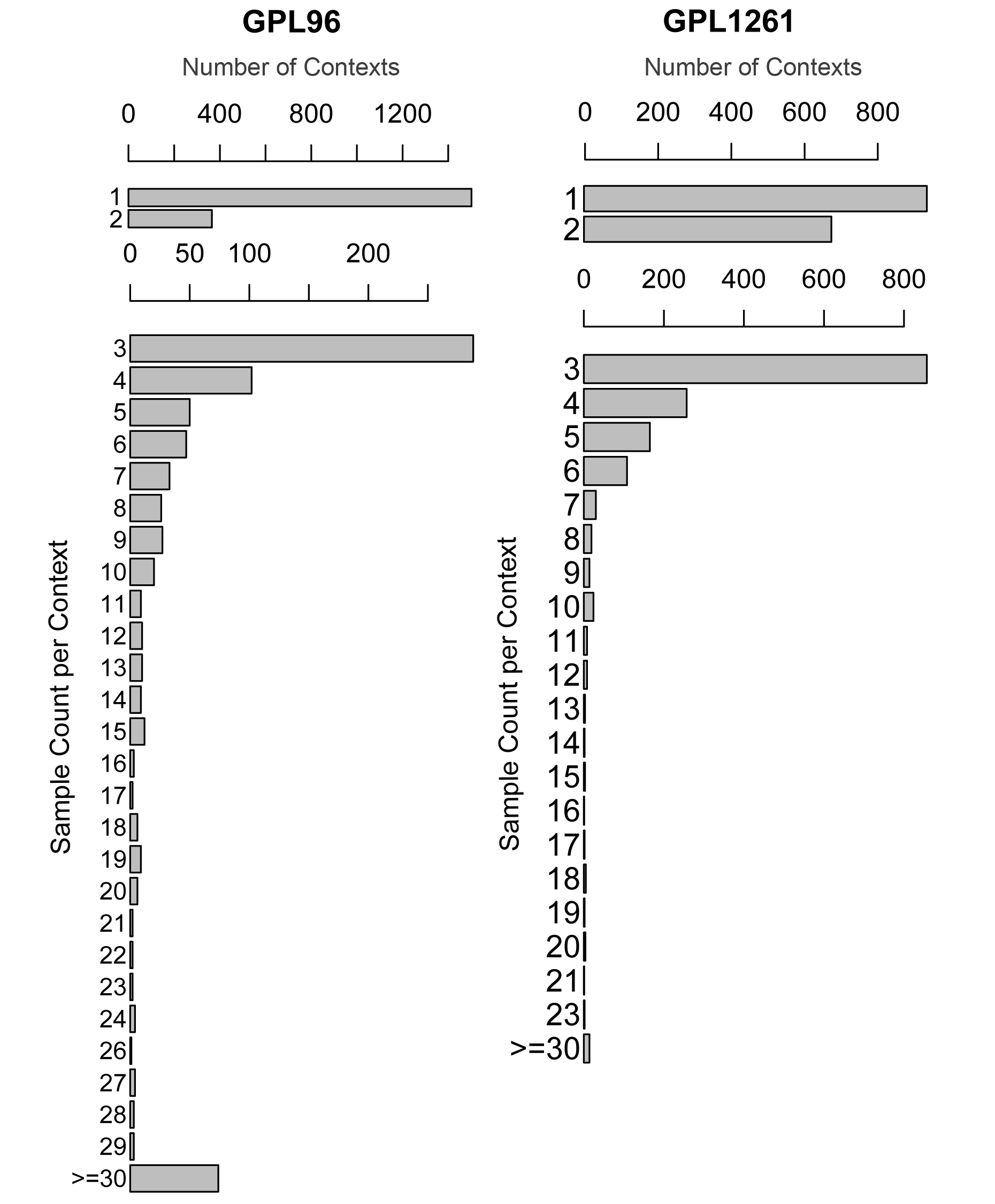
**

**Figure S1.** Distribution of the number of biological contexts with different sample counts per context in the mouse GPL1261 compendium compiled from 9,643 Affymetrix Mouse 430 2.0 samples and human GPL96 compendium compiled from 13,182 Affymetrix Human U133a samples. Different scales for contexts with counts <3 and >=3 are used to better display the tail part of the distribution.


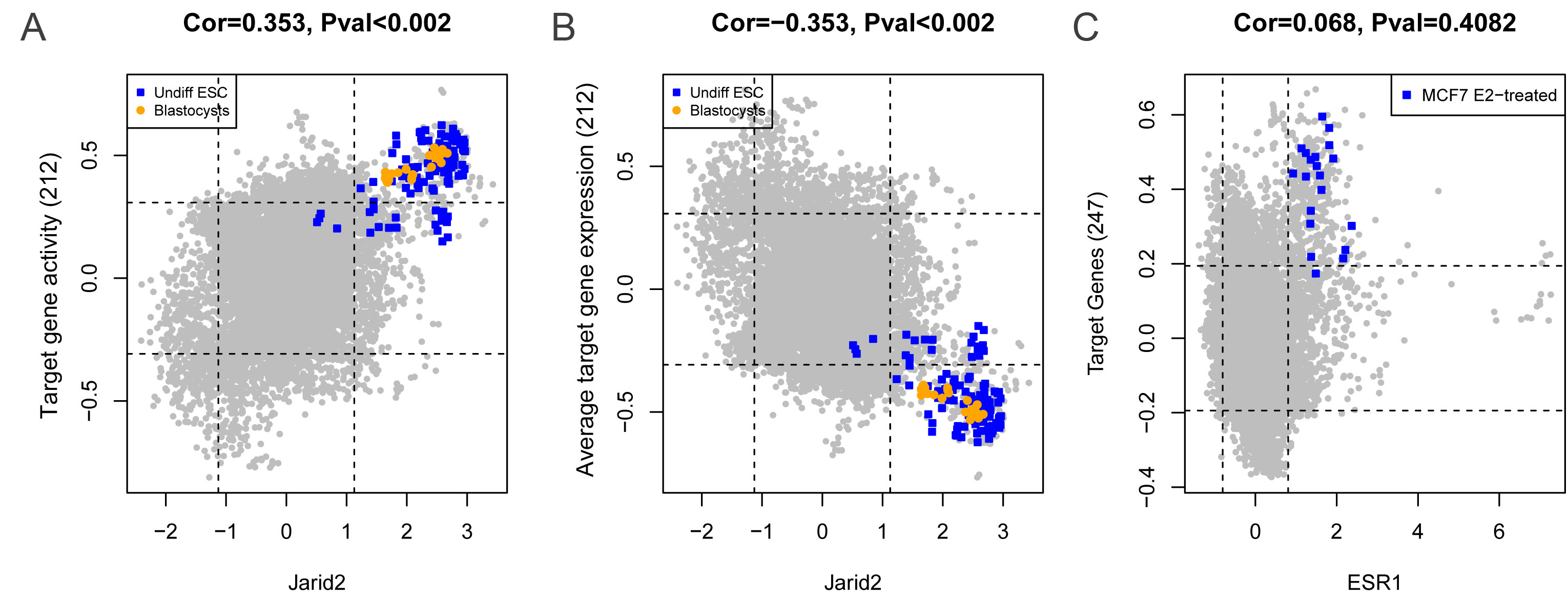


**Figure S2.** Additional ChIP-PED analysis plots. Plot of (A) *Jarid2* TF expression against target gene activity across all 9,643 samples in the mouse GPL1261 compendium, (B) *Jarid2* TF expression against average target gene expression across all 9,643 samples in the mouse GPL1261 compendium, and (C) *ESR1* TF expression against target gene activity across all samples in the 13,182 human GPL96 compendium. In (B) compared to (A), average target gene expression is plotted on the y-axis instead of target gene activity to demonstrate the difference between target gene activity and average target gene expression. Target genes in both (A) and (B) include only negatively regulated (repressed) targets of *Jarid2*. In (C), although the p-value (=0.41) for *E_TF_* and *A_TG_* correlation in *ESR1* is not significant, the *ESR1* ChIP-PED analysis is still able to recover known functional contexts, such as MCF7 cells treated with E2. “Cor.”: Pearson correlation coefficient between *E_TF_* and *A_TG_*. Contexts of interest are highlighted in color. P-values are calculated by randomly drawing pseudo-TG sets of the same size 10,000 times to construct a null correlation distribution. Dashed lines correspond to the TF+, TG+, TF-, and TG- cutoffs.

**
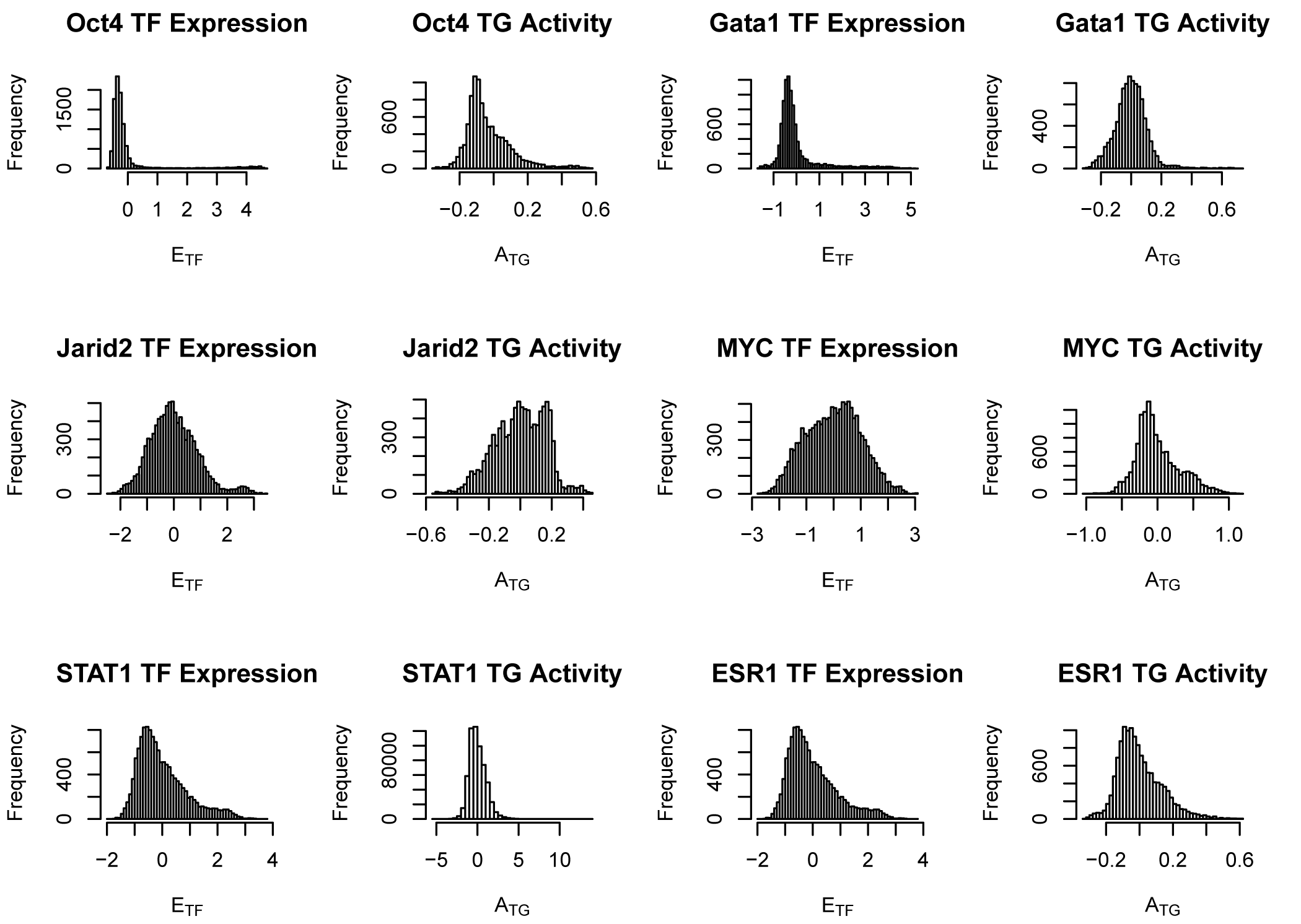
**

**Figure S3.** Histograms depicting the distribution of transcription factor (TF) expression and target gene (TG) activity. *E_TF_* is the expression of the TF and *A_TG_* is the activity of the target genes. Target genes are defined from real ChIPx and TF perturbation experiments by intersecting TF-bound genes in ChIPx data with differentially expressed genes in TF perturbation data.


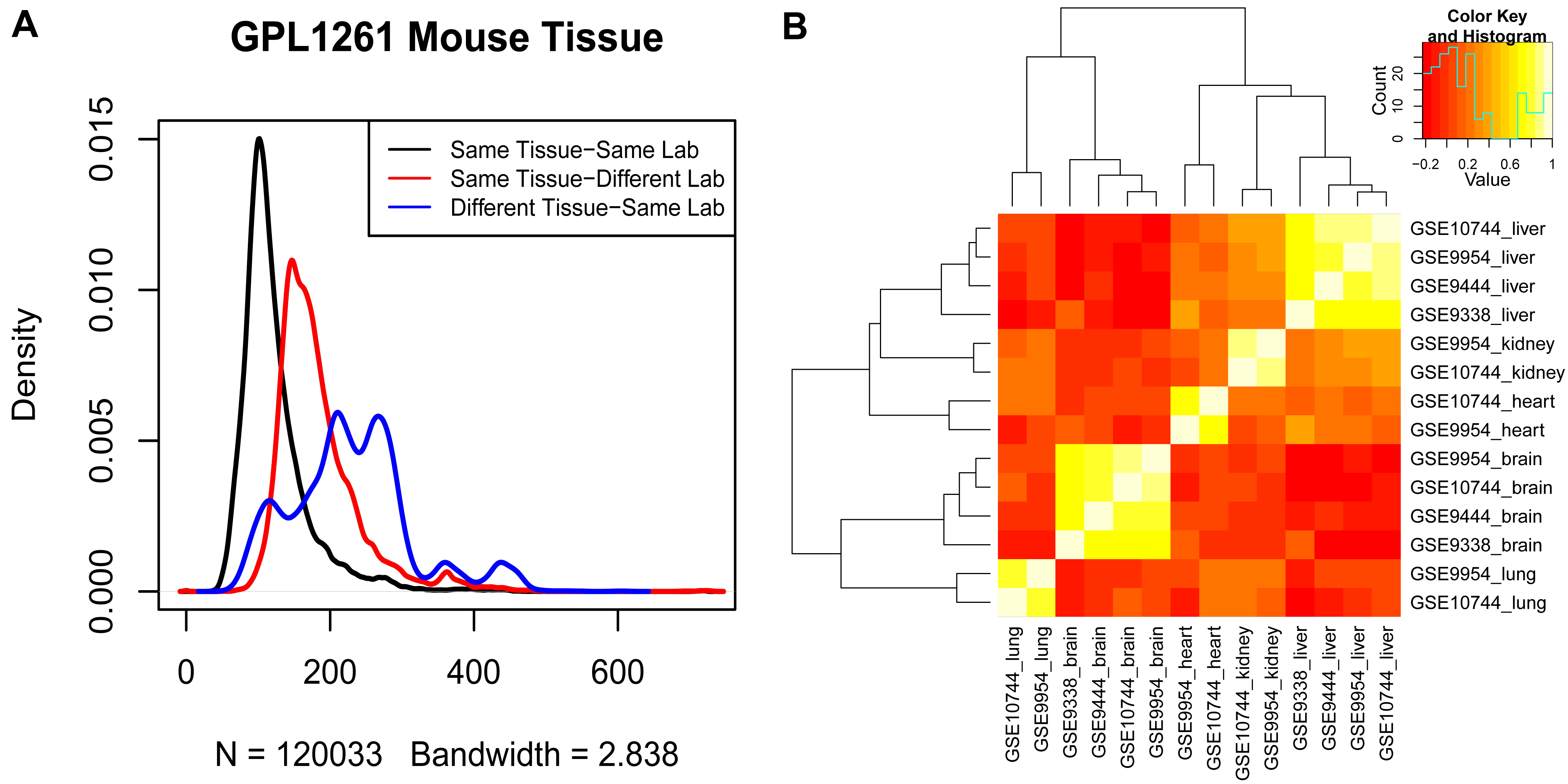


**Figure S4.** An illustration that the biological effects in the GPL1261 gene expression compendium is larger than the lab and batch effects. (A) Density plot of Euclidean distances between GPL1261 microarray expression measurements for each sample pair in the compendium belonging to three types: same tissue-same lab pairs (black), same tissue-different lab pairs (red), and different tissue-same lab pairs (blue). (B) Heatmap of tissues clustered from multiple labs, each represented by a GSE experiment ID, with multiple overlapping tissues. Results for the GPL96 compendium are similar and therefore not shown.

**
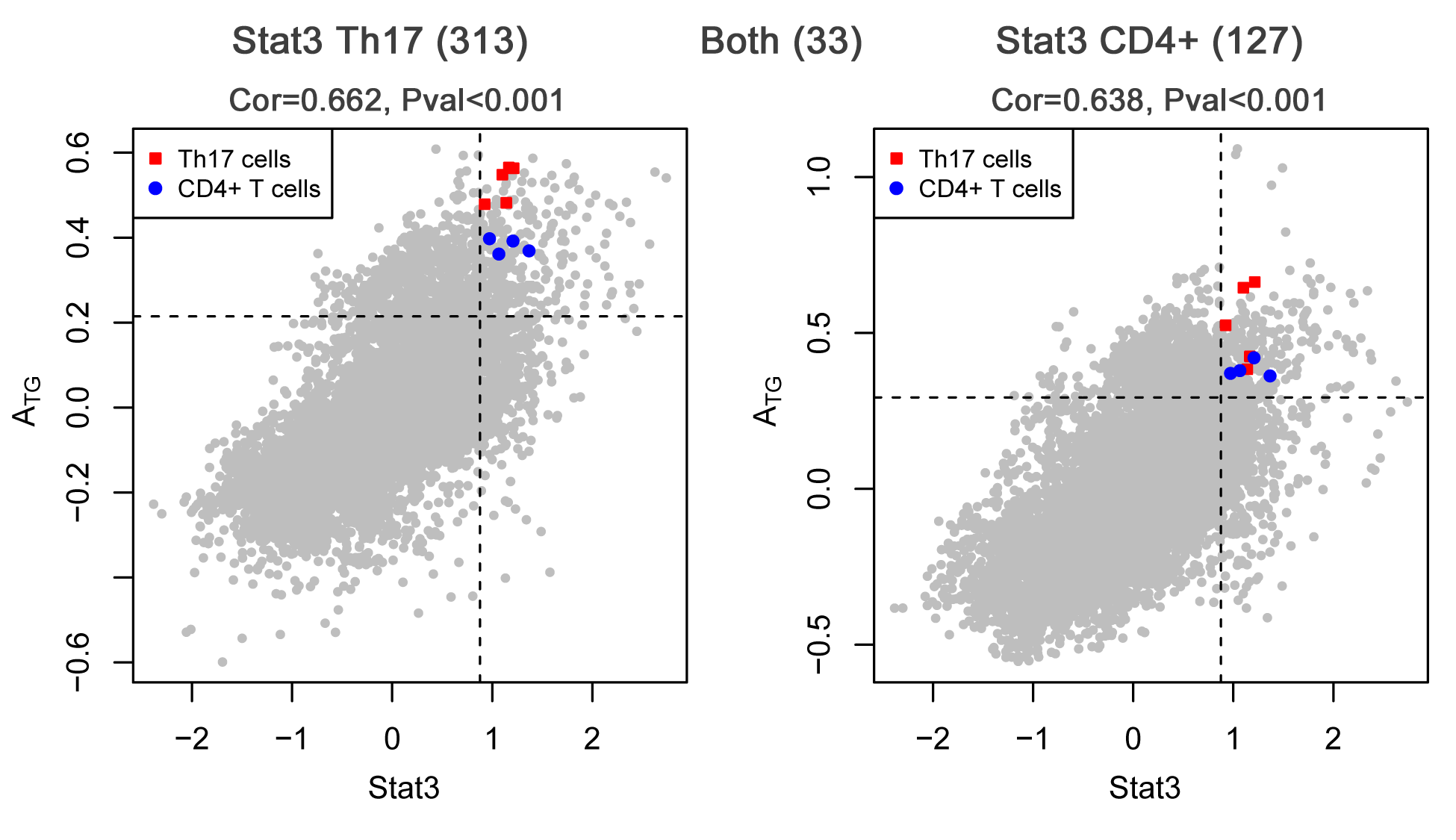
**

**Figure S5**. ChIP-PED plots of *Stat3* TF+TG+ activity derived from *Stat3* ChIP and gene expression data in Th17 cells (A) and CD4+ T cells (B). The number of target genes of *Stat3* in Th17 cells, the number of target genes of *Stat3* in CD4+ cells, and the number of target genes in common between the two cell types are shown at the top in parentheses. Although only 33 of the *Stat3* CD4+ T cell target genes and *Stat3* Th17 cell target genes overlap, both *Stat3*-active cell types, Th17 cells and CD4+ T cells, are recovered using the target genes defined from the other cell type. Significant correlation between *Stat3* TF expression and TG activity is also observed for both datasets (p-value < 0.001). Gray points are individual samples in the GPL1261 mouse compendium. P-values are calculated by randomly drawing pseudo-TG sets of the same size 10,000 times to construct a null correlation distribution. Dashed lines correspond to the TF+ and TG+ cutoffs.

**
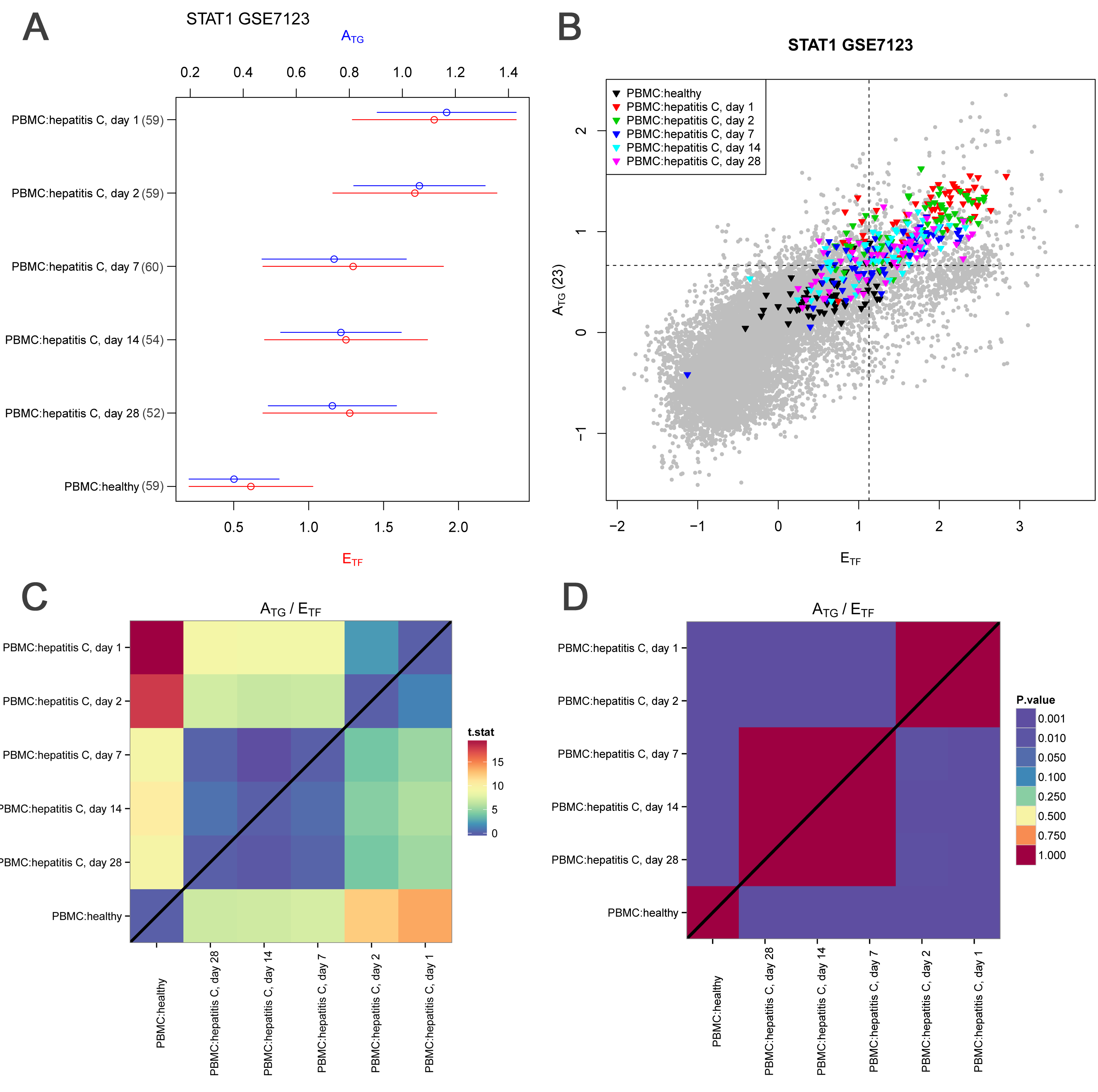
**

**Figure S6.** Follow-up ChIP-PED analysis results for PBMC hepatitis C infected contexts predicted to be enriched with *STAT1* TF+TG+ functional activity in GSE7123. In (A), *E_TF_* (red) and *A_TG_* (blue) values (mean ± one standard deviation) for each context in GSE7123 show that as more days are allowed for recovery, *E_TF_* and *A_TG_* values decrease to the baseline PBMC:healthy *E_TF_* and *A_TG_* values. Contexts are ordered from top to bottom (high to low) by the average *E_TF_* and *A_TG_* rank for each context. The number of samples in each context is shown in parentheses next to each context. Similarly in (B), a ChIP-PED plot of *E_TF_* and *A_TG_* values with each individual sample in each context highlighted in color show a decreasing trend of STAT1 functional activity with increasing recovery time. Gray points in (B) are other samples in the human GPL96 compendium and the dashed lines are the TF+ and TG+ cutoffs. T-tests comparing the mean difference of *E_TF_* and *A_TG_* values between all pair-wise combinations of contexts in GSE7123 further show that healthy PBMCs have significantly lower *E_TF_* and *A_TG_* values than hepatitis-C infected PBMCs as seen by the t-statistics (C) and p-values (D) depicted in the heatmaps. *A­_TG_* results are reported in the top left-hand half of the heatmap and *E_TF_* results are reported in the bottom right-hand half of the heatmap. T-tests are performed by subtracting the row context by the column context for *A_TG_* and the column context by the row context for *E_TF_*; e.g. the top right t.statistic (dark red) in (C) and p-value in (D) is calculated by substracting the mean *A_TG_* value of PBMC:hepatitis C, day 1 samples by the mean *A_TG_* value of PBMC:healthy samples and the bottom left t.statistic (dark orange) in (C) and p-value in (D) is calculated by subtracting the mean *E_TF_* value of PBMC:hepatitis C, day 1 samples by the mean *E_TF_* value of PMBC:healthy samples.

**
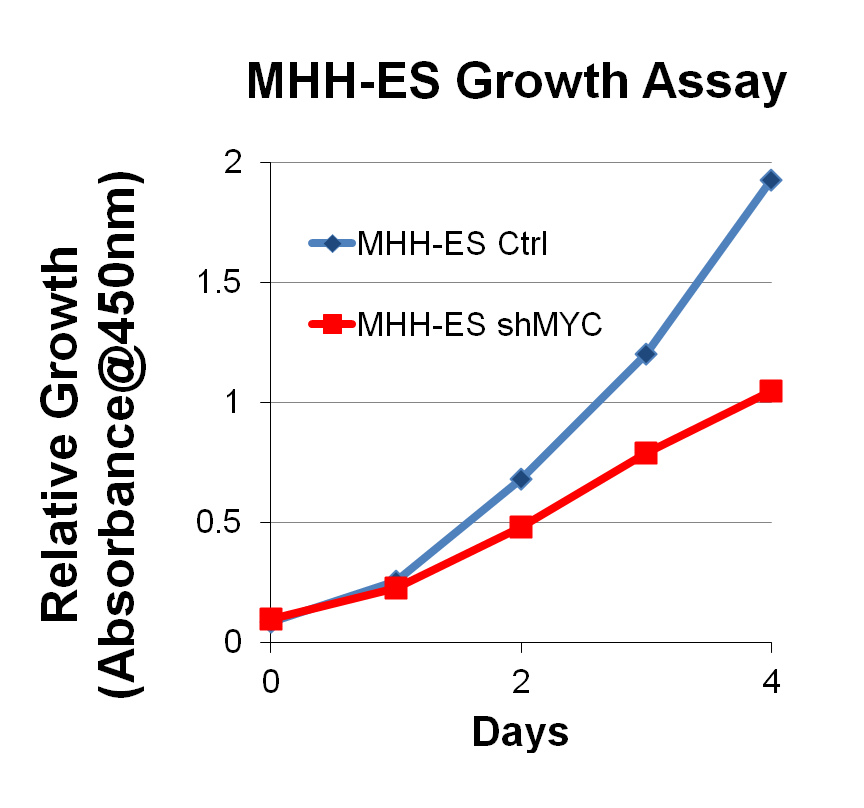
**

**Figure S7.** Decrease in proliferation of MHH-ES cells upon knockdown of c-Myc. Control and shMyc MHH-ES cells were evaluated for changes in proliferation rates by utilizing a cell viability reagent, CCK-8. 2,000 cells were initially plated into individual 96 wells and assessed daily for changes in growth and proliferation.

**
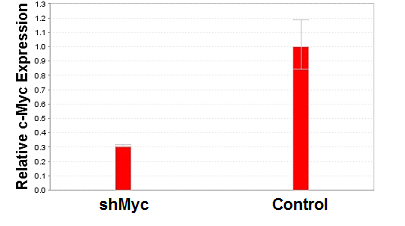
**

**Figure S8.** Relative c-Myc expression in control and c-Myc knockdown Ewing’s sarcoma cells. Quantitative real-time PCR was performed on total RNA extracted from TC71 cells expressing control or c-Myc specific shRNA. Expression levels were normalized to β-Actin, which was used as an internal loading control. Error bars correspond to one standard deviation.

**
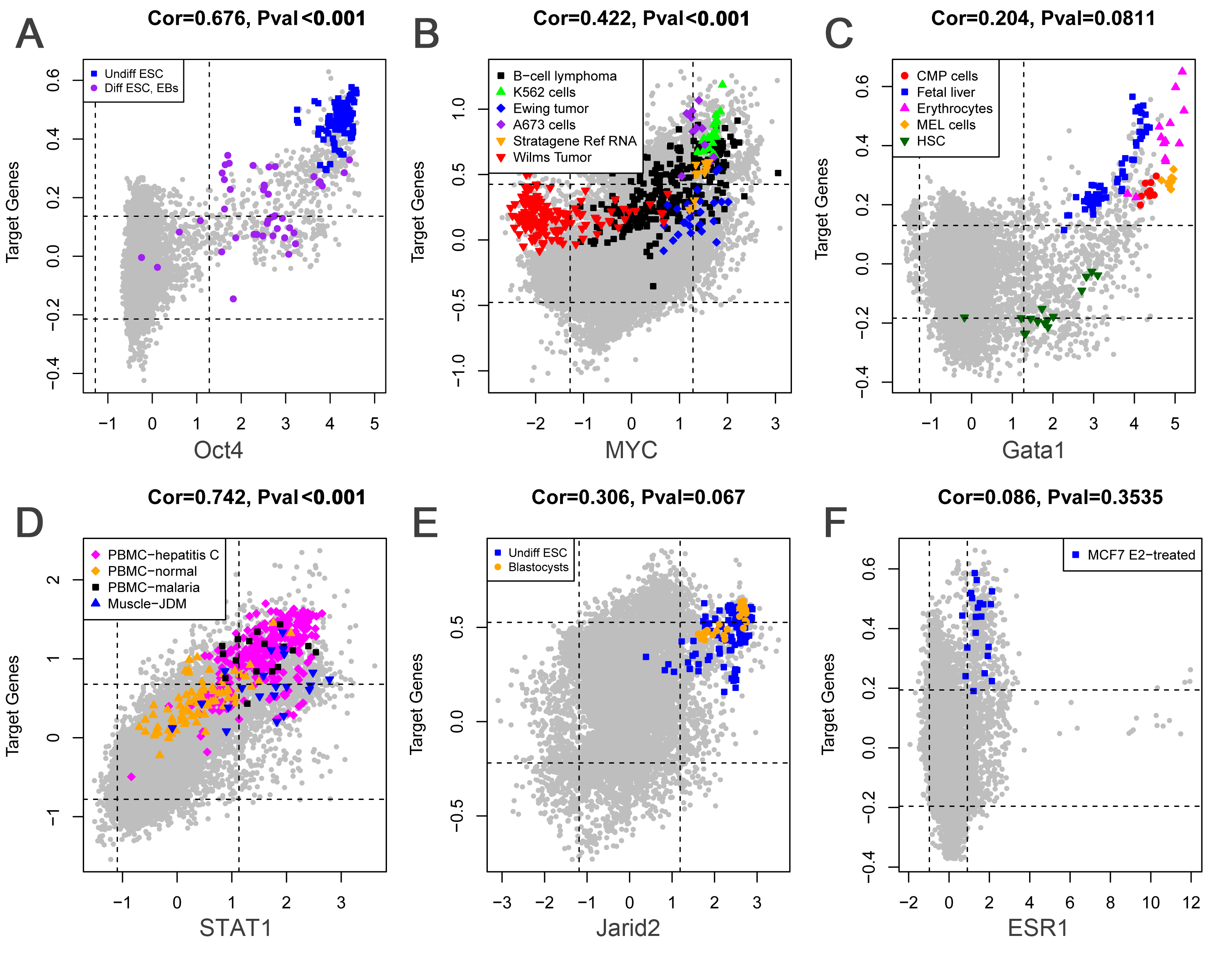
**

**Figure S9.** Plot of *Oct4* (A), *MYC* (B), *Gata1* (C), *STAT1* (D), *Jarid2* (E) and *ESR1* (F) expression against target gene activity, where TF expression and target gene activity are determined using the median probeset intensity and the median TF expression and TG activity scores. Target genes for each TF are constructed from TF-bound genes in ChIPx experiments and differentially expressed genes in TF perturbation experiments. Gray points represent each of the 9,643 samples in the compendium of gene expression profiles (in A, C, and E) built from the Affymetrix Mouse 430 2.0 arrays (GPL1261) or each of the 13,182 samples (in B, D, and F) built from the Affymetrix Human HGU133a arrays (GPL96). “Cor.”: Pearson correlation coefficient between *E_TF_* and *A_TG_*. Contexts of interest are highlighted in color. P-values are calculated by randomly drawing pseudo-TG sets of the same size 10,000 times to construct a null correlation distribution. Dashed lines correspond to the TF+, TG+, TF-, and TG- cutoffs.

**
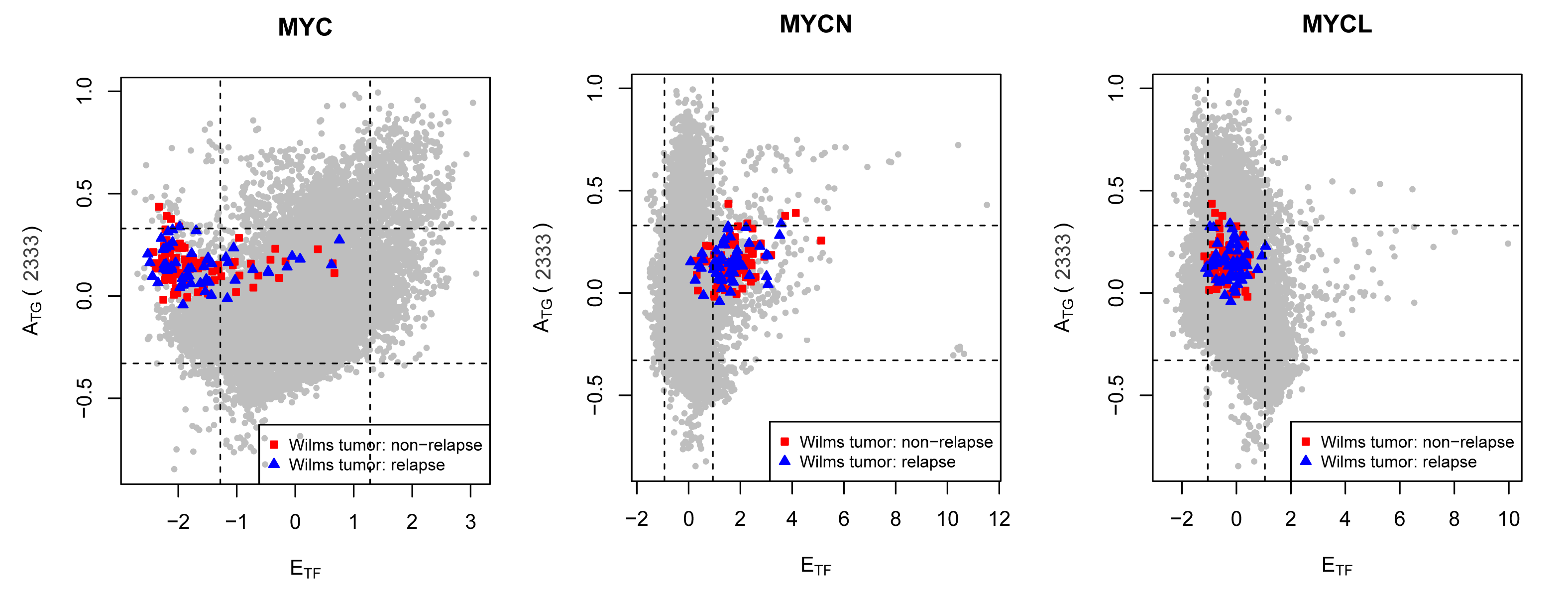
**

**Figure S10.** Plot of *MYC*, *MYCN*, and *MYCL* TF expression (*E_TF_*) against *MYC* target gene activity (*A_TG_*). In Wilms tumor samples (highlighted in color), *MYC* TF-TG+ regulatory activity is predicted to be significantly enriched (left). Significantly enriched functional activity (TF+TG+) of possible compensating *MYC*-homologous TFs – *MYCN, MYCL* – is also not detected in Wilms tumors (middle-right). The lack of significance for *MYCN*, even though *MYCN* is highly expressed, is primarily because the proportion of samples with *A_TG_* score above the TG+ cutoff is not large enough compared to the proportion of background TF+TG+ samples, which suggests that we may not have enough power to detect significant *MYCN* functional activity through *MYC* target genes. Despite this, the Wilms tumor samples that fall within TF-TG+ region in the *MYC* plot were also found in the TF+TG+ region in the *MYCN* plot, therefore *MYCN* may still be a candidate that could compensate for *MYC* regulatory activity in Wilms tumor. In contrast, *MYCL* has a low *E_TF_* score, thus it is less likely that *MYCL* compensates for *MYC* regulatory activity in Wilms tumor. Gray points represent each of the 13,182 samples from Affymetrix Human HGU133a arrays (GPL96). Number of *MYC* target genes is displayed in parentheses on the y-axis. Dashed lines correspond to the TF+, TF-, TG+, and TG- cutoffs.
